# Evinacumab in severe hypertriglyceridemia with or without lipoprotein lipase pathway mutations: a phase 2 randomized trial

**DOI:** 10.1038/s41591-023-02222-w

**Published:** 2023-03-06

**Authors:** Robert S. Rosenson, Daniel Gaudet, Christie M. Ballantyne, Seth J. Baum, Jean Bergeron, Erin E. Kershaw, Patrick M. Moriarty, Paolo Rubba, David C. Whitcomb, Poulabi Banerjee, Andrew Gewitz, Claudia Gonzaga-Jauregui, Jennifer McGinniss, Manish P. Ponda, Robert Pordy, Jian Zhao, Daniel J. Rader

**Affiliations:** 1 Metabolism and Lipids Unit, Mount Sinai Heart, Icahn School of Medicine at Mount Sinai, New York, NY USA; 2 Clinical Lipidology and Rare Lipid Disorders Unit, Department of Medicine, Université de Montréal Community Gene Medicine Center, and ECOGENE-21 Clinical and Translational Research Center, Chicoutimi, Quebec Canada; 3 Department of Medicine, Baylor College of Medicine, Houston, TX USA; 4 Excel Medical Clinical Trials and Department of Integrated Medical Sciences, Charles E Schmidt College of Medicine, Florida Atlantic University, Boca Raton, FL USA; 5 Departments of Laboratory Medicine and of Medicine, Centre Hospitalier Universitaire de Québec-Université Laval, Québec, Québec Canada; 6 Division of Endocrinology, Department of Medicine, University of Pittsburgh, Pittsburgh, PA USA; 7 Division of Clinical Pharmacology, University of Kansas Medical Center, Kansas City, KS USA; 8 Department of Clinical Medicine and Surgery, Federico II University of Naples, Naples, Italy; 9 Division of Gastroenterology, Hepatology and Nutrition, University of Pittsburgh, Pittsburgh, PA USA; 10 Regeneron Pharmaceuticals, Tarrytown, NY USA; 11 Department of Genetics and Department of Medicine, Perelman School of Medicine, University of Pennsylvania, Philadelphia, PA USA

**Keywords:** Fat metabolism, Dyslipidaemias

## Abstract

Severe hypertriglyceridemia (sHTG) is an established risk factor for acute pancreatitis. Current therapeutic approaches for sHTG are often insufficient to reduce triglycerides and prevent acute pancreatitis. This phase 2 trial (NCT03452228) evaluated evinacumab (angiopoietin-like 3 inhibitor) in three cohorts of patients with sHTG: cohort 1, familial chylomicronemia syndrome with bi-allelic loss-of-function lipoprotein lipase (LPL) pathway mutations (*n* = 17); cohort 2, multifactorial chylomicronemia syndrome with heterozygous loss-of-function LPL pathway mutations (*n* = 15); and cohort 3, multifactorial chylomicronemia syndrome without LPL pathway mutations (*n* = 19). Fifty-one patients (males, *n* = 27; females, *n* = 24) with a history of hospitalization for acute pancreatitis were randomized 2:1 to intravenous evinacumab 15 mg kg^−1^ or placebo every 4 weeks over a 12-week double-blind treatment period, followed by a 12-week single-blind treatment period. The primary end point was the mean percent reduction in triglycerides from baseline after 12 weeks of evinacumab exposure in cohort 3. Evinacumab reduced triglycerides in cohort 3 by a mean (s.e.m.) of −27.1% (37.4) (95% confidence interval −71.2 to 84.6), but the prespecified primary end point was not met. No notable differences in adverse events between evinacumab and placebo treatment groups were seen during the double-blind treatment period. Although the primary end point of a reduction in triglycerides did not meet the prespecified significance level, the observed safety and changes in lipid and lipoprotein levels support the further evaluation of evinacumab in larger trials of patients with sHTG. Trial registration number: ClinicalTrials.gov NCT03452228.

## Abstract (web summary)

The potential of evinacumab, a monoclonal antibody targeting angiopoietin-like 3, for reducing triglyceride levels was tested in patients with severe hypertriglyceridemia due to differing genetic etiologies.

## Main

sHTG is a well-established risk factor for acute pancreatitis (AP) and is considered causal in 10% of cases^1^. The 2018 American Heart Association/American College of Cardiology guidelines on the management of blood cholesterol define severe hypertriglyceridemia as ≥500 mg dl^−1^ (ref. ^2^). Similarly, the National Lipid Association defines very-high triglycerides (highest classification possible) as ≥500 mg dl^−1^ (refs. ^2,3^). Furthermore, within the US Food and Drug Administration prescribing information of triglyceride-lowering drugs, sHTG is commonly defined as ≥500 mg dl^−1^ (ref. ^4^). In the United States, the prevalence of sHTG (≥500 mg dl^−1^) is reported to be 1.7%^5^. In a retrospective cohort study including US adults (*n* = 7,119,195), the overall annualized incidence rate of AP was 0.08% and increased with increasing triglyceride levels (0.07%, triglycerides <200 mg dl^−1^; 1.21%, triglycerides >1,000 mg dl^−1^)^6^. Furthermore, for example, for patients with one or ≥2 AP events at baseline, the overall annualized incidence rate of AP was found to increase to 10.16% and 29.98%, respectively^6^. Patients with sHTG-related AP often have recurrent attacks requiring repeat hospital admissions and have worse outcomes than non-hypertriglyceridemia-related AP^7^, including an increased odds ratio for chronic morbidity and mortality^8^.

Patients with sHTG typically have chylomicronemia, most commonly caused by multiple triglyceride-elevating genetic variants exacerbated by lifestyle, comorbid diseases and/or medications^7^. This polygenic disorder is referred to as multifactorial chylomicronemia syndrome (MCS). Rarely, patients with sHTG have chylomicronemia that is monogenic in origin, arising due to loss-of-function (LOF) mutations in genes encoding LPL or other genes of the LPL pathway, including the genes encoding apolipoprotein (APO) A5 (*APOA5*), APOC2 (*APOC2*), glycosylphosphatidylinositol anchored high-density lipoprotein binding protein 1 (*GPIHBP1*) and lipase maturation factor 1 (*LMF1*), resulting in familial chylomicronemia syndrome (FCS)^7,9^. The development of AP, which is often recurrent, is the most important clinical complication of MCS and FCS^10^. Current therapeutic approaches to sHTG include weight loss, dietary counseling, fibrates and omega-3 fatty acid products; however, these approaches are often insufficient to reduce triglycerides and prevent AP in a substantial number of patients^2,11,12^.

Angiopoietin-like 3 (ANGPTL3) is an important regulator of lipoprotein metabolism, acting as an inhibitor of LPL and endothelial lipase (EL)^12–14^. Individuals with LOF variants in the gene encoding ANGPTL3 (*ANGPTL3*) have markedly reduced triglycerides, suggesting that it could be a therapeutic target for lowering triglycerides by increasing LPL activity^15–17^. Evinacumab is a fully human monoclonal antibody that inhibits ANGPTL3 (refs. ^14–16,18,19^) and previous studies have assessed its efficacy and safety in patients with hypertriglyceridemia^20,21^. In individuals with hypertriglyceridemia, a peak median reduction in triglycerides of 81.8% at day 4 was observed with intravenous (i.v.) evinacumab 10 mg kg^−1^ (versus a 20.6% reduction with placebo), with effects seen up to day 43 (ref. ^21^). In individuals with triglycerides >1,000 mg dl^−1^, wide-ranging triglyceride reductions were observed with subcutaneous evinacumab 250 mg kg^−1^ and i.v. evinacumab 20 mg kg^−1^, ranging from 0.9% to 93.2% on day 3, sustained until day 22 in most individuals^21^. In the present study, we evaluated the safety and efficacy of evinacumab in patients with sHTG and a history of hospitalization for AP.

## Results

### Disposition of patients and treatments

**Fig. 1:**
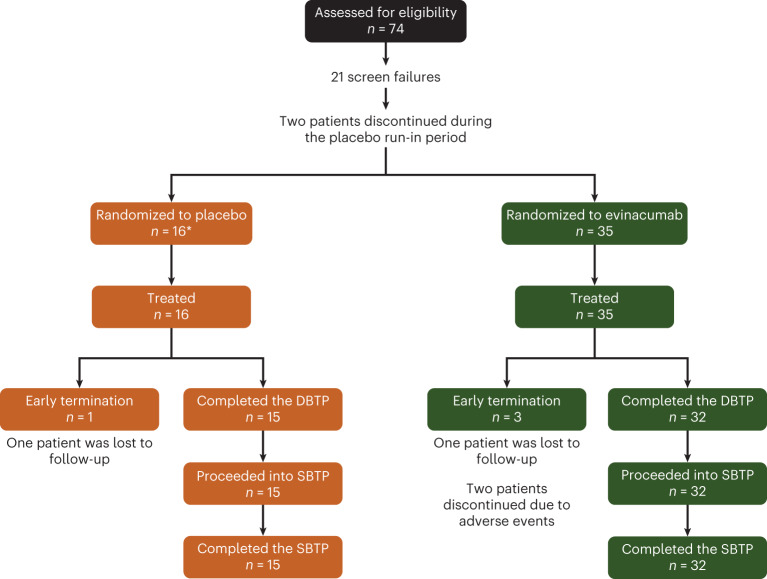
CONSORT diagram. *A total of 17 patients were randomized to placebo; however, one patient who failed screening was erroneously randomized and was withdrawn from the study.

**Fig. 2:**
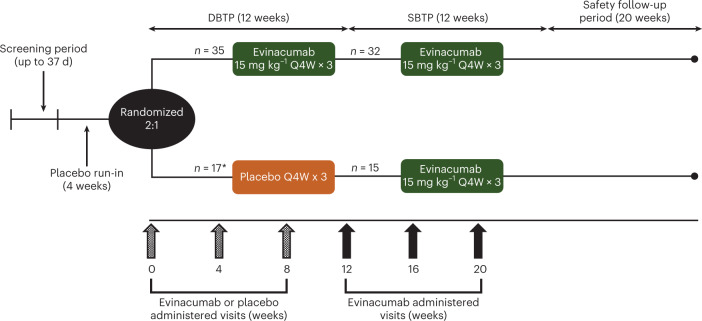
Study design. *One patient was randomized but not treated.

**Extended Data Fig. 1:**
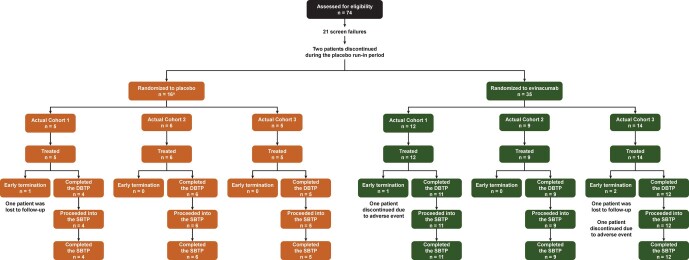
Patient disposition by cohort. ^a^In total, 17 patients were randomized to placebo. However, one patient who failed screening was erroneously randomized and was withdrawn from the study. DBTP, double-blind treatment period; SBTP, single-blind treatment period.

A total of 74 patients were screened, of whom 21 were screen failures. A further two patients discontinued during the placebo run-in period. Thus, during the double-blind treatment period (DBTP), 51 patients were treated (Fig. 1 and Extended Data Fig. 1). A summary of patient genotype by actual cohort is detailed in Extended Data Table 1 and genetic variants identified in the overall patient cohort are detailed in Supplementary Table 1. Three evinacumab-treated patients (adverse events (*n* = 2); lost to follow-up (*n* = 1)) and one placebo-treated patient (lost to follow-up) did not complete the DBTP (for an overview of the study design refer to Fig. 2). The adverse events leading to treatment discontinuation during the DBTP for evinacumab-treated patients were AP (severe serious adverse event, considered related to study treatment) and influenza-like illness (non-serious, moderate in severity, considered unrelated to study treatment). A total of 47 patients (double-blind (DB) evinacumab, *n* = 32; DB placebo, *n* = 15) entered the single-blind treatment period (SBTP).

### Patient demographics and baseline characteristics

Patient demographics were generally well balanced between the placebo and evinacumab groups in the DBTP and SBTP (Table 1 and Extended Data Table 2, respectively). As expected, baseline median fasting triglycerides were higher in cohort 1 versus cohorts 2 and 3 (Table 2 and Extended Data Table 3). Baseline lipid-lowering therapy data are shown in Table 1. Most patients were receiving oral anti-diabetic therapy at baseline (66.7% and 66.0% for the DBTP and SBTP, respectively). During the DBTP, mean (s.d.) change in weight from baseline to week 12 ranged from −0.14 (2.1) kg to +0.83 (1.7) kg across all cohorts. Similarly, during the SBTP, mean (s.d.) change in weight from baseline to week 24 ranged from −0.14 (3.2) kg to +2.2 (3.9) kg across all cohorts.

**Table 1:** Demographics and baseline characteristics of patients in the DBTP

	Cohort 1		Cohort 2		Cohort 3	
	Placebo i.v. Q4W (*n* = 5)	Evinacumab i.v. 15 mg kg^−1^ Q4W (*n* = 12)	Placebo i.v. Q4W (*n* = 6)	Evinacumab i.v. 15 mg kg^−1^ Q4W (*n* = 9)	Placebo i.v. Q4W (*n* = 5)	Evinacumab i.v. 15 mg kg^−1^ Q4W (*n* = 14)
Age (years) mean (s.d.)	43.2 (15.7)	51.3 (9.4)	52.8 (13.5)	48.7 (10.3)	41.2 (7.8)	46.1 (11.0)
Sex (male) *n* (%)	4 (80.0)	6 (50.0)	2 (33.3)	6 (66.7)	3 (60.0)	6 (42.9)
Race, *n* (%)						
White	4 (80.0)	11 (91.7)	5 (83.3)	7 (77.8)	3 (60.0)	11 (78.6)
Black or African American	0	0	0	1 (11.1)	0	0
Asian	0	1 (8.3)	0	1 (11.1)	1 (20.0)	3 (21.4)
Other	1 (20.0)	0	1 (16.7)	0	1 (20.0)	0
Ethnicity, *n* (%)						
Hispanic or Latino	0	2 (16.7)	1 (16.7)	1 (11.1)	1 (20.0)	1 (7.1)
Not Hispanic or Latino	5 (100)	10 (83.3)	5 (83.3)	8 (88.9)	4 (80.0)	13 (92.9)
BMI (kg m^−2^) mean (s.d.)	26.6 (4.1)	26.8 (5.2)	27.9 (5.6)	31.5 (4.3)	30.0 (1.9)	28.9 (5.0)
History of AP, *n* (%)	5 (100)	12 (100)	6 (100)	9 (100)	5 (100)	14 (100)
Time from the most recent occurrence of AP (years) mean (s.d.)^a^	5.5 (7.8)	8.5 (9.6)	1.9 (1.1)	3.9 (3.8)	1.8 (1.6)	3.0 (4.6)
Coronary heart disease, *n* (%)	1 (20.0)	1 (8.3)	2 (33.1)	1 (11.1)	1 (20.0)	5 (35.7)
Concomitant LLTs, *n* (%)	3 (60.0)	6 (50.0)	6 (100)	9 (100)	4 (80.0)	10 (71.4)
Fibrates	2 (40.0)	5 (41.7)	5 (83.3)	8 (88.9)	4 (80.0)	8 (57.1)
Statins	2 (40.0)	3 (25.0)	3 (50.0)	6 (66.7)	3 (60.0)	9 (64.3)
High-intensity statins	1 (20.0)	1 (8.3)	1 (16.7)	4 (44.4)	1 (20.0)	9 (64.3)
Nicotinic acid and derivatives	0	1 (8.3)	0	3 (33.3)	0	0
Other^b^	3 (60.0)	5 (41.7)	5 (83.3)	7 (77.8)	0	8 (57.1)
≥2 LLTs	2 (40.0)	5 (41.7)	6 (100.0)	7 (77.8)	2 (40.0)	8 (57.1)
≥3 LLTs	2 (40.0)	3 (25.0)	2 (33.3)	6 (66.7)	0 (0.0)	7 (50.0)
≥4 LLTs	0 (0.0)	0 (0.0)	0 (0.0)	0 (0.0)	0 (0.0)	1 (7.1)
≥5 LLTs	0 (0.0)	0 (0.0)	0 (0.0)	0 (0.0)	0 (0.0)	0 (0.0)
Concomitant antihyperglycemic drugs, *n* (%)	2 (40.0)	5 (41.7)	3 (50.0)	7 (77.8)	5 (100)	12 (85.7)
Biguanides (metformin)	1 (20.0)	5 (41.7)	3 (33.3)	4 (44.4)	3 (60.0)	8 (57.1)
Insulin (fast acting)	2 (40.0)	0	2 (33.3)	4 (44.4)	1 (20.0)	6 (42.9)
Insulin (long acting)	2 (40.0)	2 (16.7)	1 (16.7)	3 (33.3)	1 (20.0)	4 (28.6)
SGLT2 inhibitors	0 (0.0)	0 (0.0)	1 (16.7)	4 (44.4)	3 (60.0)	3 (21.4)
GLP-1 inhibitors	0 (0.0)	0 (0.0)	0 (0.0)	1 (11.1)	0 (0.0)	2 (14.3)

^a^Time from diagnosis to study randomization.

^b^Includes omega-3-acid ethyl ester, omega-3 fatty acids, eicosapentaenoic acid ethyl ester, ezetimibe, fish oil, combination of docosahexaenoic acid, eicosapentaenoic acid and fish oil, eicosapentaenoic acid and omega-3 triglycerides.

BMI, body mass index; GLP-1, glucagon-like peptide-1; LLT, lipid-lowering therapy; Q4W, every 4 weeks; SGLT2, sodium glucose co-transporter 2.

**Table 2:** Change in lipid/lipoprotein parameters from baseline to week 12 in the DBTP

	Cohort 1		Cohort 2		Cohort 3	
	Placebo i.v. Q4W (*n* = 5)	Evinacumab i.v. 15 mg kg^−1^ Q4W (*n* = 12)	Placebo i.v. Q4W (*n* = 6)	Evinacumab i.v. 15 mg kg^−1^ Q4W (*n* = 9)	Placebo i.v. Q4W (*n* = 5)	Evinacumab i.v. 15 mg kg^−1^ Q4W (*n* = 14)
**Fasting triglycerides, mg** **dl**^**−1**^						
Baseline, median (Q1:Q3)	3,918.3 (3,122.3:3,931.3)	3,140.7 (2,713.0:3,921.0)	1,351.5 (768.7:4,010.3)	1,238.0 (1,020.3:2,341.0)	1,030.7 (1,021.7:1,495.7)	1,917.3 (1,196.0:2,607.3)
Percent change from baseline, median (Q1:Q3)	−22.9 (−34.5:−12.5)	−27.7 (−68.5:2.2)	9.4 (0.2:25.7)	−64.8 (−84.5:−41.8)	80.9 (27.2:112.9)	−81.7 (−90.5:−21.7)
*P* value versus placebo		0.9495		0.0076		0.0418
**Total cholesterol, mg** **dl**^**−1**^						
Baseline, mean (s.d.)	372.2 (107.7)	363.5 (115.0)	220.3 (127.4)	257.3 (136.5)	230.0 (60.4)	319.6 (149.3)
Percent change from baseline, median (Q1:Q3)	−12.8 (−17.1:−1.0)	−33.3 (−58.6:−26.1)	7.9 (−11.4:23.3)	−31.1 (−60.6:−29.5)	43.3 (16.9:47.8)	−34.6 (−62.6:5.9)
*P* value versus placebo		0.0157		0.0216		0.0787
**Non-HDL-C, mg** **dl**^**−1**^						
Baseline, mean (s.d.)	355.6 (107.6)	345.0 (117.0)	201.5 (128.0)	220.0 (151.3)	208.6 (57.5)	296.0 (148.7)
Percent change from baseline, median (Q1:Q3)	−15.2 (−17.9:−2.7)	−34.2 (−61.0:−25.9)	8.0 (−8.2:25.6)	−31.0 (−47.6:−3.1)	48.4 (15.1:55.5)	−38.5 (−66.7:8.9)
*P* value versus placebo		0.0074		0.0677		0.1016
**Remnant cholesterol, mg** **dl**^**−1**^						
Baseline, mean (s.d.)	348.0 (98.9)	319.3 (127.6)	158.2 (139.3)	174.1 (159.5)	141.5 (46.0)	253.9 (158.4)
Percent change from baseline, median (Q1:Q3)	−17.5 (−18.8:−3.6)	−37.5 (−67.7:−25.4)	24.2 (8.2:38.7)	−62.8 (−86.2:−18.3)	76.9 (23.3:104.2)	−79.0 (−90.1:9.3)
*P* value versus placebo		0.0133		0.0157		0.0602
**LDL-C, mg** **dl**^**−1**^^**a**^						
Baseline, mean (s.d.)	26.0 (20.3)	21.6 (15.3)	43.3 (24.8)	45.7 (25.5)	43.4 (16.8)	62.6 (63.1)
Percent change from baseline, median (Q1:Q3)	8.6 (−11.9:47.5)	25.0 (40.0:63.2)	−15.5 (−56.7:11.5)	26.5 (12.9:39.9)	−40.0 (−44.6:4.2)	32.0 (−34.8:130.9)
*P* value versus placebo		0.9384		0.0735		0.1700
**HDL-C, mg** **dl**^**−1**^						
Baseline, mean (s.d.)	16.8 (3.4)	18.7 (4.1)	18.8 (2.9)	37.6 (43.8)	20.0 (4.6)	23.4 (9.4)
Percent change from baseline, mean (s.d.)	10.9 (25.6)	−18.5 (37.3)	−17.4 (30.1)	−26.6 (27.0)	11.0 (37.4)	−7.5 (26.4)
Mean percent difference versus placebo (95% CI)		−33.0 (−79.5:13.5)		1.87 (−22.6:26.3)		−18.0 (−56.2:20.2)
*P* value versus placebo		0.1476		0.8706		0.3269
**Total ApoB, mg** **dl**^**−1**^						
Baseline, mean (s.d.)	73.3 (32.3)	74.1 (29.9)	85.5 (23.9)	90.8 (14.8)	86.3 (8.5)	120.9 (50.1)
Percent change from baseline, mean (s.d.)	5.3 (13.0)	−16.4 (26.9)	−3.4 (22.6)	−11.1 (14.7)	20.6 (21.0)	−11.6 (23.1)
Mean percent difference versus placebo (95% CI)		−21.2 (−46.5:4.0)		−8.2 (−30.0:13.6)		−27.0 (−56.7:2.8)
*P* value versus placebo		0.0919		0.4302		0.0722
**ApoB100, mg** **dl**^**−1**^						
Baseline, mean (s.d.)	61.2 (25.8)	61.2 (32.4)	82.4 (24.0)	84.4 (10.4)	76.8 (14.2)	114.4 (55.8)
Percent change from baseline, median (Q1:Q3)	−1.0 (−6.9:26.0)	−26.1 (−43.7:4.3)	−5.4 (−32.1:20.4)	−9.9 (−19.1:7.9)	19.4 (−4.1:45.2)	−12.0 (−17.0:−1.6)
*P* value versus placebo		0.1704		0.9485		0.0745
**ApoB48, mg** **dl**^**−1**^						
Baseline, mean (s.d.)	12.0 (8.2)	12.9 (7.8)	3.1 (2.0)	9.4 (8.4)	9.5 (8.3)	6.1 (3.7)
Percent change from baseline, median (Q1:Q3)	2.3 (−32.7:89.2)	−26.4 (−49.9:13.2)	65.1 (21.2:191.6)	−45.9 (−86.4:−15.4)	41.5 (−4.0:194.7)	−77.0 (−86.9:17.7)
*P* value versus placebo		0.6477		0.0332		0.1066
**ApoC3, mg** **dl**^**−1**^						
Baseline, mean (s.d.)	42.7 (21.1)	40.2 (11.2)	26.5 (11.0)	45.4 (26.2)	48.4 (8.0)	46.4 (27.9)
Percent change from baseline, mean (s.d.)	1.6 (23.3)	−33.7 (33.6)	47.1 (50.7)	−33.6 (59.6)	30.7 (32.9)	−54.9 (44.2)
Mean percent difference versus placebo (95% CI)		−35.7 (−84.4:13.1)		−64.1 (−133.9:5.8)		−83.9 (−137.0:30.7)
*P* value versus placebo		0.1361		0.0688		0.0046
**ApoA1, mg** **dl**^**−1**^						
Baseline, mean (s.d.)	97.6 (16.1)	103.3 (18.4)	101.3 (17.1)	114.1 (28.2)	126.4 (39.6)	122.6 (19.3)
Percent change from baseline, mean (s.d.)	−6.0 (8.0)	−31.5 (9.1)	−2.6 (20.2)	−28.8 (14.1)	−2.8 (18.6)	−24.4 (17.6)
Mean percent difference versus placebo (95% CI)		−25.4 (−37.1:−13.7)		−23.6 (−43.4:−3.9)		−21.8 (−45.9:2.2)
*P* value versus placebo		0.0005		0.0229		0.0717
**Lp(a), nmol** **l**^**−1**^						
Baseline, median (Q1:Q3)	8.0 (6.0:21.0)	16.0 (8.0:23.0)	10.0 (7.0:10.0)	12.0 (9.0:76.0)	11.0 (6.0:12.0)	20.0 (9.0:42.0)
Percent change from baseline, median (Q1:Q3)	100.0 (−38.1:233.3)	12.5 (−12.5:75.0)	17.9 (−31.3:65.0)	0.0 (−8.3:16.8)	0.0 (−14.3:33.3)	−14.3 (−31.0:55.6)
*P* value versus placebo		0.5045		0.8081		0.6114

Post hoc nominal *P* values are provided for descriptive purposes only. Apo, apolipoprotein; Lp(a), lipoprotein (a); Q1, first quartile; Q3, third quartile.

^a^LDL-C concentrations were determined by ultracentrifugation.

### Treatment exposure

Treatment exposure during the DBTP was generally consistent across the evinacumab and placebo groups. The mean (s.d.) number of infusions was almost identical for the evinacumab and placebo groups (2.8 (0.6) infusions); the mean (s.d.) duration of study drug exposure was also almost identical between the evinacumab and placebo groups (11.4 (2.4) and 11.4 (2.3) weeks, respectively). Doses were missed by two patients in the placebo group (at week 4 due to hospitalization for abdominal pain (*n* = 1); at week 8 due to hospitalization for AP (*n* = 1)). With the exception of three patients who discontinued treatment after receiving the first dose on study day 1, no evinacumab-treated patient missed doses of study drug during the DBTP.

Mean (s.d.) duration of study drug exposure during the SBTP was similar for the DB evinacumab (11.5 (1.4) weeks) and DB placebo (12.1 (1.0) weeks) groups. Evinacumab doses were missed by three patients during the SBTP. Two patients were unable to attend the scheduled visit due to an adverse event; one patient had a visit outside of the window, so the site was advised not to dose the patient and to wait for the next visit (<2 weeks later).

### Efficacy of triglyceride lowering

The prespecified primary end point of this trial was the least squares mean percent reduction in triglycerides from baseline after 12 weeks of evinacumab exposure (combination of DBTP and SBTP) in cohort 3. The mean (s.e.m.) percent reduction in triglycerides from baseline in cohort 3 was –27.1% (37.4) (95% confidence interval (CI) –71.2 to 84.6); however, the log-transformed triglyceride values were not normally distributed, making use of mean percent change in triglyceride levels a less-than-ideal end point. Therefore, we also performed a post hoc analysis using median percent reductions in triglyceride values (results presented below).

**Fig. 3:**
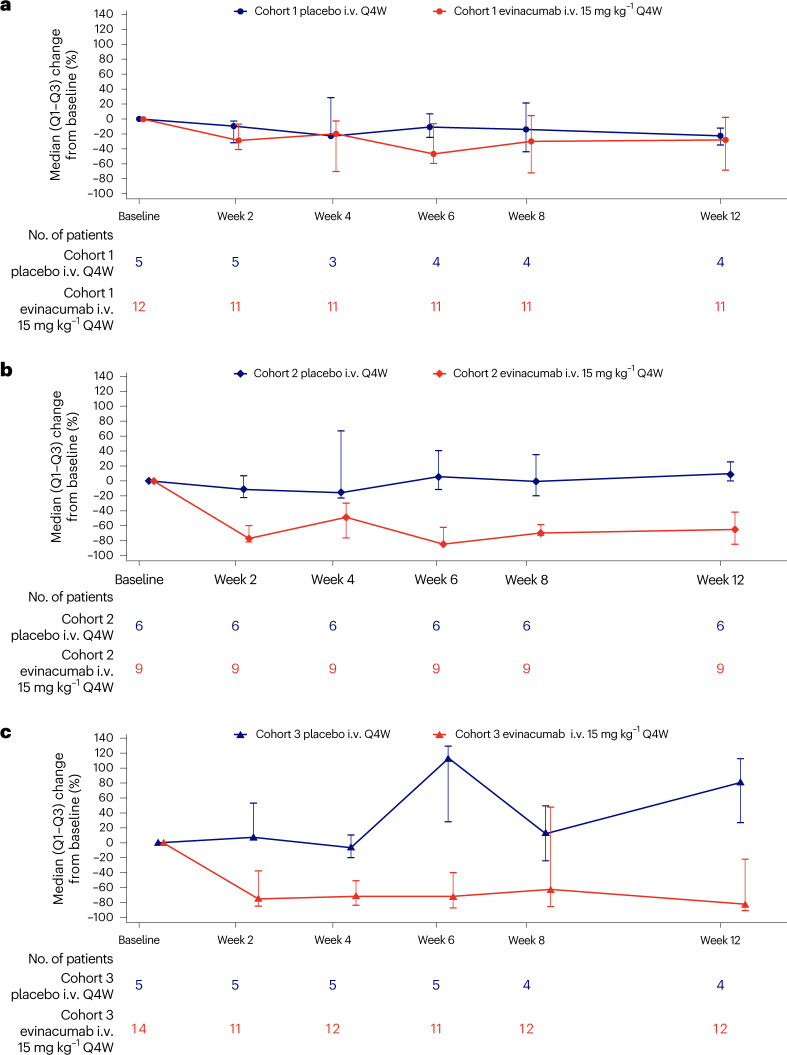
Median percent change in fasting triglycerides. **a**–**c**, Median (Q1 to Q3) percent change in fasting triglycerides from baseline to week 12 by cohort in the DBTP (exploratory end points). **a**, data for cohort 1; **b**, data for cohort 2; **c**, data for cohort 3.

During the DBTP, the three cohorts were heterogeneous in the triglyceride-lowering response observed. Notably, the LPL-deficient cohort 1 exhibited substantially less response to evinacumab than the other two cohorts (Fig. 3 and Table 2; exploratory end point). At week 12, the cohort 1 median percent triglyceride reduction in treated versus placebo individuals was −27.7% versus −22.9% (absolute median change of −753 versus −782 mg dl^−1^; *P* = 0.9495) respectively, whereas in cohort 2 the response was –64.8% versus +9.4% (absolute median change of −675 versus +118 mg dl^−1^; *P* = 0.0076) and in cohort 3 was –81.7% versus +80.9% (absolute median change of −1,141 versus +805 mg dl^−1^; *P* = 0.0418). The triglyceride-lowering effect with evinacumab (and the lack of effect on triglycerides in cohort 1) was observed to be maintained through to week 24 during the SBTP (Extended Data Table 3; exploratory end point).

A post hoc analysis of the median percent reduction in triglycerides from baseline following 12 weeks of evinacumab exposure was also conducted. In cohort 3, the median percent reduction in triglycerides was –68.8%; the absolute median change in cohort 3 was –905 mg dl^−1^.

### Efficacy on other lipid/lipoprotein parameters

Evinacumab was effective in reducing non-high-density lipoprotein cholesterol (non-HDL-C) in all three cohorts, including cohort 1, at week 12 (the end of the DBTP; Table 2; exploratory end point). Median percent reductions in non-HDL-C with evinacumab treatment versus placebo were –34.2% versus –15.2% in cohort 1 (*P* = 0.0074), –31.0% versus +8.0% in cohort 2 (*P* = 0.0677); and –38.5% versus +48.4% in cohort 3 (*P* = 0.1016). Similar effects were seen with evinacumab treatment versus placebo on remnant cholesterol (–37.5% versus –17.5%; *P* = 0.0133 (cohort 1); –62.8% versus +24.2%; *P* = 0.0157 (cohort 2); –79.0% versus +76.9%; *P* = 0.0602 (cohort 3); Table 2; exploratory end point). There was a trend toward increased low-density lipoprotein cholesterol (LDL-C) with evinacumab versus placebo in this sHTG cohort: +25.0% versus +8.6%, *P* = 0.9384 (cohort 1); +26.5% versus –15.5%, *P* = 0.0735 (cohort 2); +32.0% versus –40.0%; *P* = 0.1700 (cohort 3; Table 2; exploratory end point). Evinacumab treatment was associated with a trend toward lowering of total ApoB levels versus placebo (–16.4% versus +5.3%; *P* = 0.0919 (cohort 1); –11.1% versus –3.4%; *P* = 0.4302 (cohort 2); –11.6% versus +20.6%; *P* = 0.0722 (cohort 3)), with similar results on ApoB100 and ApoB48 (Table 2; exploratory end points). Notably, evinacumab treatment resulted in reduction in plasma ApoC3 levels that was substantial in cohort 3 (–33.7% versus +1.6%; *P* = 0.1361 (cohort 1); –33.6% versus +47.1%; *P* = 0.0688 (cohort 2); –54.9% versus +30.7%; *P* = 0.0046 (cohort 3); Table 2; exploratory end points). While HDL-C levels were not substantially affected by evinacumab treatment, there was a substantial reduction in ApoA1 levels (Table 2; exploratory end point). Overall, changes in lipid/lipoprotein parameters observed during the DBTP were maintained during the SBTP (Extended Data Table 3; exploratory end points).

### Assessment of evinacumab pharmacokinetics/pharmacodynamics

**Extended Data Fig. 2:**
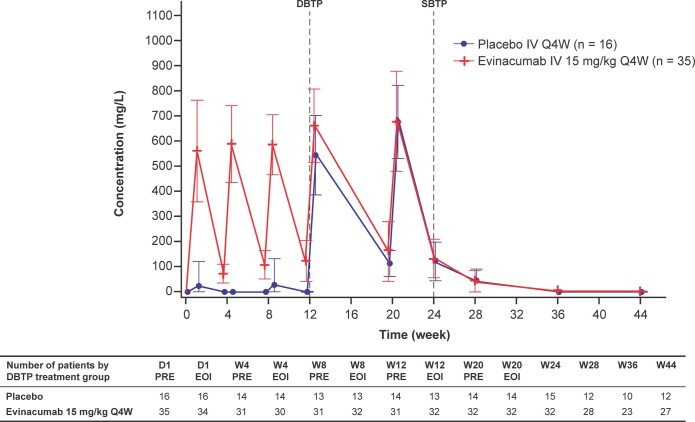
Mean (SD) of total serum evinacumab concentrations by time and treatment group. The DBTP includes all measurements until week 12 pre-dose; the SBTP includes all measurements from week 12 EOI. During the SBTP, all patients received IV evinacumab 15 mg/kg Q4W. D, day; DBTP, double-blind treatment period; EOI, end of infusion; IV, intravenous; PRE, pre-dose; Q4W, every 4 weeks; SBTP, single-blind treatment period; SD, standard deviation; W, week.

**Extended Data Fig. 3:**
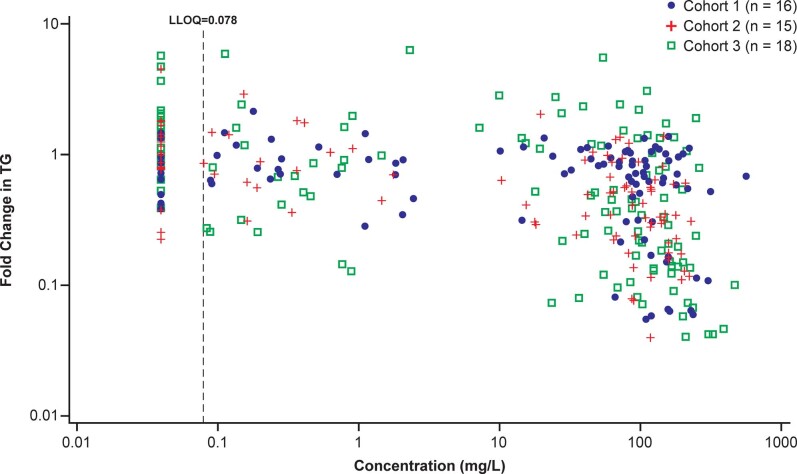
Individual log-scaled fold change from baseline in triglycerides versus log-scaled trough concentrations of total evinacumab. Fold change is derived as (individual TG value at each timepoint)/(baseline TG value). Concentrations below the LLOQ were set to LLOQ/2. BLQ, below the limit of quantification; LLOQ, lower limit of quantification; N, number of patients; TG, triglyceride.

Pharmacokinetic data analyzed in all individuals showed that steady-state concentrations of total evinacumab were reached by the end of the DBTP (three doses), with mean steady-state evinacumab trough concentrations (C_trough_) fluctuating between 120 and 160 mg l^−1^ through the end of the SBTP (Extended Data Fig. 2). Substantial inter-patient variability in evinacumab serum C_trough_ was observed, with lower evinacumab exposure associated with low or no triglyceride-lowering response (Extended Data Fig. 3). The variability in response, associated with low drug levels, introduced skew into the primary efficacy end point.

### Safety and tolerability during the placebo-controlled period

In the DBTP, treatment-emergent adverse events (TEAEs) occurred in 71.4% and 68.8% of evinacumab- and placebo-treated patients, respectively (secondary end point). Common TEAEs occurring in >5% of patients in any treatment group are detailed in Table 3; those occurring more frequently in the evinacumab versus placebo group included abdominal pain, headache, constipation, abdominal discomfort, increase in alanine aminotransferase/aspartate aminotransferase (defined as three times the upper limit of normal), back pain, contusion, dizziness, herpes zoster and sinusitis. Nasopharyngitis, AP and type 2 diabetes mellitus occurred less frequently in the evinacumab than in the placebo group. Serious TEAEs were reported in four (11.4%) patients in the evinacumab group (abdominal pain (*n* = 1), AP (*n* = 3); two events of AP in one patient were considered related to study treatment) and three (18.8%) patients in the placebo group (AP (*n* = 2), abdominal pain (*n* = 1); none was considered related to study treatment). TEAEs leading to discontinuation occurred in two (5.7%) evinacumab-treated patients (AP (*n* = 1); influenza-like illness (*n* = 1)) and 0% of placebo-treated patients. There were no deaths in either treatment group. Corresponding TEAE data for the combined SBTP and off-drug follow-up period are presented in Supplementary Table 2.

**Table 3:** Summary of TEAEs in any treatment group during the DBTP

TEAEs (*n* (%) of patients)	Placebo i.v. Q4W (*n* = 16)	Evinacumab 15 mg kg^−1^ i.v. Q4W (*n* = 35)
Patients with at least one TEAE	11 (68.8)	25 (71.4)
Patients with at least one serious TEAE	3 (18.8)	4 (11.4)
Patients with at least one TEAE resulting in discontinuation of treatment	0	2 (5.7)
Patients with any TEAE resulting in death	0	0
TEAEs occurring in ≥2 patients in any group		
Abdominal pain	2 (12.5)	5 (14.3)
Headache	1 (6.3)	4 (11.4)
Constipation	0	3 (8.6)
AP	2 (12.5)	3 (8.6)
Abdominal discomfort	0	2 (5.7)
Alanine aminotransferase increased	0	2 (5.7)
Aspartate aminotransferase increased	0	2 (5.7)
Back pain	0	2 (5.7)
Contusion	0	2 (5.7)
Dizziness	0	2 (5.7)
Herpes zoster	0	2 (5.7)
Nasopharyngitis	1 (6.3)	2 (5.7)
Sinusitis	0	2 (5.7)
Type 2 diabetes mellitus	1 (6.3)	2 (5.7)

### Imaging

^18^F-FDG-positron-emission tomography (PET)/computed tomography (CT) and magnetic resonance imaging (MRI) did not identify patients with subclinical signs of pancreatitis at baseline (Extended Data Table 4). Imaging values evaluated were in range with typical physiological levels. No clinically relevant imaging changes in the pancreas were observed in patients who were treated with evinacumab (Extended Data Table 5; secondary end point). There was a large distribution of baseline hepatic fat fractions ranging from healthy levels (3%) to substantially elevated (38%). No changes in hepatic fat fractions were observed at week 24 compared to baseline in patients treated with evinacumab (Extended Data Table 6; exploratory end point).

### AP events during this study

Through the course of this 44-week study (including off-drug washout), a total of 25 AP events were reported (Supplementary Table 3; exploratory end point). During the DBTP, five AP events were reported by five patients (evinacumab group (*n* = 3); placebo group (*n* = 2)). Within the evinacumab group, one patient in cohort 1 experienced an AP event on study day 54 (resolved within 4 d) and two patients in cohort 3 experienced AP events 2 and 12 d following the first evinacumab dose, respectively. All three evinacumab-treated patients had triglyceride levels >1,000 mg dl^−1^ at the time of, or immediately before, their AP episode. During the 12-week SBTP active treatment period, seven AP events in five patients were reported and, during the 12-week off-drug follow-up period, 13 AP events in ten patients were reported. In the combined SBTP and off-drug follow-up period, most AP events occurred >4 weeks after the last evinacumab dose when triglycerides had increased back toward pre-treatment levels and evinacumab concentrations had decreased to near baseline levels (Supplementary Table 3 and Supplementary Fig. 1).

During the combined SBTP and off-drug follow-up period, triglyceride measurements were available for 15 of 20 AP events; of the 15 AP events with available triglyceride levels, most patients had triglycerides >500 mg dl^−1^ at the time of, or immediately before, the AP episode. As AP events were reported by investigative sites but not independently adjudicated, events did not always meet typical diagnostic criteria, such as the international consensus Atlanta classification^22^. The available data, including laboratory and imaging results, are summarized in Supplementary Table 3.

## Discussion

As severely elevated levels of serum concentrations are an established risk factor for AP, effective triglyceride-lowering therapies are required to improve patient outcomes. In this phase 2, randomized, placebo-controlled study in individuals with sHTG (fasting serum triglycerides >500 mg dl^−1^ at screening; medical history of fasting triglycerides ≥1,000 mg dl^−1^) and with a history of hospitalization for AP, the prespecified primary end point of percent change in mean triglycerides was not met; however, triglycerides were not normally distributed and a post hoc analysis of the median percent change in triglycerides suggested triglyceride reduction with evinacumab, except in those patients with FCS due to LPL deficiency. Although treatment with evinacumab in patients with FCS demonstrated a non-substantial increase in LDL-C, substantial reductions in non-HDL-C, remnant cholesterol and other triglyceride associated measures and non-substantial reductions in total ApoB and ApoB100 were observed, indicating an overall reduction in atherogenic lipids.

The treatment response to evinacumab was highly variable, in part influenced by the molecular etiology of sHTG. For example, during the placebo-controlled period cohort 1 (consisting of patients with FCS due to bi-allelic mutations in known FCS genes) had no reduction in triglycerides, while reductions were observed in both cohort 2 (only one FCS gene mutation) and cohort 3 (no identified FCS gene mutations). The reduced response in cohort 1 during the DBTP may be due to the markedly diminished LPL activity expected in these patients. Patients in cohort 3, observed to have a median reduction in triglycerides of –81.7% with evinacumab, are likely to have high polygenic risk scores for hypertriglyceridemia, whereas patients in cohort 2 may have a combination of rare variants in the context of a high polygenic risk score background^7,23^.

The inter-individual variability in evinacumab exposure likely contributed to the observed variability in triglyceride-lowering response. Other sources of variability in triglyceride responses may include inconsistent adherence to diet and exercise, known effects of some background lipid-lowering therapies and the use of a single post-treatment assessment of triglycerides (in contrast to three measurements used to determine baseline values).

The decreases in remnant cholesterol and ApoB48 support a role for evinacumab in promoting chylomicron remnant catabolism. Parallel decreases in ApoC3 and ApoB100, despite an increase in LDL-C, are consistent with the broader role of evinacumab in triglyceride-rich lipoprotein (TRL) cholesterol^14,24^, with the increase in LDL-C likely due to enhanced conversion of very-low-density lipoprotein particles to low-density lipoprotein particles. The reductions in HDL-C and ApoA1 after treatment with evinacumab were anticipated based on the known effects of ANGPTL3 inhibition on de-repression of EL activity leading to increased clearance of HDL^25^.

ApoC3 is an important regulator of triglyceride metabolism via inhibition of LPL-mediated hydrolysis of TRLs to smaller remnant particles and impaired removal of TRL remnants^17,26^. The mechanisms by which ApoC3 impairs lipolysis of TRLs have been partly elucidated and include weakened binding of TRLs to the negatively charged capillary endothelium where LPL is present and inhibiting LPL activation by displacing the LPL activator ApoC2 from the surface of the TRL particle^27–29^. Patients with sHTG typically have increased ApoC3 levels due to elevation of triglyceride-rich lipoproteins carrying ApoC3. Here we find that evinacumab treatment in patients with sHTG substantially reduced plasma ApoC3 levels. It is interesting to speculate that the ApoC3 reduction with evinacumab may have contributed to the triglyceride reduction in this population. The reduction in ApoC3 that we observed was likely to be at least in part secondary to the reduction in TRLs brought about by evinacumab; however, given the reduction of ApoC3 levels even in cohort 1 and out of proportion to the triglyceride reduction, we speculate that the unmasking of EL activity by inhibition of ANGPTL3 may play a role in the reduction of plasma ApoC3, given that HDL is a reservoir for ApoC3. This is consistent with the considerable reduction in plasma ApoA1 levels with evinacumab, which notably were out of proportion to the modest and non-substantial effect on HDL-C levels.

Treatment with evinacumab was generally well tolerated. During the DBTP, there were no clinically meaningful differences in TEAE frequency between the evinacumab and placebo groups. TEAEs also occurred in a similar proportion of patients in the DB evinacumab and DB placebo groups during the SBTP. Notably, treatment with evinacumab had no effect on liver transaminases or hepatic fat as measured by MRI in contrast to a recent report of an antisense oligonucleotide targeted to ANGPTL3 (ref. ^30^).

Over the duration of the study (including the off-drug follow-up period) a total of 25 AP events were reported. During the DBTP, all patients had serum triglycerides >1,000 mg dl^−1^ at the time of, or just before, their AP event, irrespective of their treatment group. Overall, proximate evinacumab levels suggest that drug concentrations were likely inadequate to provide a sustained reduction in triglycerides or blunt the anticipated post-prandial rise in triglycerides that can trigger an AP event. Future studies with evinacumab in patients with sHTG focusing on AP as the primary end point are needed. Furthermore, additional studies with other evinacumab dosing regimens, in part due to the lack of sustained evinacumab concentrations observed in the current trial, are needed to explore the association between the reduction in triglycerides and with the incidence of AP events in this sHTG patient population.

Limitations of this trial include the small number of patients studied, the relatively short duration of treatment, the variability of serum triglycerides and the variability in evinacumab exposure. These limit the assessment of the long-term safety and efficacy of evinacumab in patients with sHTG.

sHTG-associated AP is a substantial source of morbidity, mortality and reduced quality of life as well as a financial burden to the health systems caring for these patients. Neither diet nor current pharmacological therapies have substantially addressed these consequences of sHTG. In this study, while the prespecified primary end point of mean percent change in triglyceride was not met, a post hoc analysis of median triglyceride suggested reductions in fasting triglycerides following evinacumab, except in patients with FCS lacking functional LPL. These data support the critical need to further assess the effects of evinacumab in subjects with sHTG, especially in those with a history of sHTG-associated AP.

## Methods

This phase 2 study (ClinicalTrials.gov identifier NCT03452228) was conducted at 17 sites across four countries. The first patient was enrolled on 7 June 2018 and the last patient was enrolled on 8 July 2019. The study protocol was approved by institutional review boards (IRBs) and/or ethics committees (Quorum Review, Comitato Etico dell Universita, Policlinico Umberto I di Roma, North West – Greater Manchester South Research Ethics Committee, The University of Pennsylvania IRB, The University of Texas Institutional Review Board, Western IRB, Human Research Protection Program, The University of Kansas Medical Center and Copernicus IRB). The study was conducted in accordance with ethical principles originating from the Declaration of Helsinki and was consistent with International Conference on Harmonization/Good Clinical Practices and applicable regulatory requirements. All participants provided written informed consent. The principal investigators and sponsor designed the study protocol and selected participating sites. Monitoring and site supervision were performed by a contract research organization with oversight by the sponsor. The first author wrote all drafts of the manuscript. All authors had access to the data, participated in revisions and vouch for the accuracy and completeness of data and adherence to the protocol.

### Study design and treatment

Adults aged 18 to 75 years with sHTG (fasting serum triglycerides >500 mg dl^−1^ at screening on two separate occasions; documented medical history of fasting triglycerides ≥1,000 mg dl^−1^) with a history of hospitalization for AP were enrolled based on genotype according to the presence of LOF mutations in LPL pathway genes. Cohort 1 consisted of patients with FCS (with known bi-allelic LOF mutations in *APOA5*, *APOC2*, *GPIHBP1*, *LMF1* or *LPL*); cohort 2 consisted of patients with MCS (with known heterozygous LOF mutations in *APOA5*, *APOC2*, *GPIHBP1*, *LMF1* or *LPL)*; and cohort 3 consisted of patients with MCS and without LPL pathway mutations. Initially patients were enrolled into the aforementioned cohorts based on available genotype information from the patient’s medical history at screening. All patients were subsequently exome sequenced and analyzed by the Regeneron Genetics Center (Regeneron Pharmaceuticals). At screening, 11 patients were missing genotype information; based on the exome sequencing, three of these were subsequently assigned to cohort 1 and eight were assigned to cohort 2. In addition, one patient from original cohort 3 was withdrawn from the study before dosing as they did not meet the eligibility criteria. Thus, the final ‘actual cohort’ assignments for the purpose of analysis were cohort 1, *n* = 17; cohort 2, *n* = 15; and cohort 3, *n* = 19. The full list of patient eligibility criteria is provided in the Supplementary Information.

Patients in each cohort were randomized 2:1 to receive either i.v. evinacumab 15 mg kg^−1^ every 4 weeks or matching placebo. The study comprised a screening phase, a 4-week single-blind placebo run-in, a 12-week DBTP, a 12-week SBTP and a 20-week off-drug observation phase (Fig. 2). During the SBTP, all patients received i.v. evinacumab 15 mg kg^−1^ every 4 weeks.

The primary end point of the study was to determine the intra-patient percent change in mean serum triglycerides from baseline following 12 weeks of evinacumab treatment in cohort 3 patients (the 12 weeks of treatment encompassed a combination of the DBTP and SBTP). Cohort 3 was prespecified as the analysis population for the primary end point analysis, as this group of patients had an intact LPL pathway and would thus be expected to respond optimally to evinacumab treatment. To reduce the variability of baseline triglyceride measurements for patients randomized to evinacumab in the DBTP, baseline was defined as the geometric mean of all available triglyceride results at days –28, –14 and 1. Similarly, for those switching from placebo to evinacumab in the SBTP, baseline was defined as the geometric mean of all available triglyceride results at weeks 6, 8 and 12. The percent change in other lipid/lipoprotein parameters from baseline to weeks 12 and 24 were also evaluated.

### Lipid/lipoprotein measurements

All blood sampling for the determination of lipid parameters were determined under fasting conditions (at least 8 h of fasting). Triglycerides and total cholesterol were assessed by an enzymatic colorimetric assay run on a Beckman–Coulter analyzer. HDL-C was determined by precipitation, which involved precipitating all non-HDL-C using 50 kDa dextran sulfate with magnesium ions as the precipitating agent, followed by the determination of HDL-C in the supernatant using an adapted method for determining total cholesterol on a Beckman–Coulter analyzer. LDL-C was determined by ultracentrifugation; after separation of the very-low-density lipoprotein/chylomicron sub-fraction by ultracentrifugation, LDL-C was determined as the cholesterol in the infranatant (performed by an enzymatic colorimetric assay) minus HDL-C. ApoB and ApoA1 were assessed by nephelometry using a Siemens BNII nephelometer. In addition, serial ultracentrifugation was performed to separate lipoprotein subfractions (chylomicrons, very-low-density lipoprotein, intermediate-density lipoprotein, LDL and HDL) and lipids (triglycerides, cholesterol and phospholipids) and proteins (for example, ApoB, ApoA1, ApoC2, ApoC3 and ApoC5) were measured in the fractions by established methods.

### Exome sequencing and analysis

Genomic DNA was extracted from peripheral blood samples and submitted for whole exome sequencing at the Regeneron Genetics Center (RGC). Briefly, 1 μg of genomic DNA was fragmented and prepared for exome capture with a custom reagent kit from Kapa Biosystems. Samples were captured using the NimbleGen SeqCap VCRome 2.1 exome target design and sequenced using 75-bp paired-end sequencing on an Illumina HiSeq 2500 with v.4 chemistry. Following sequencing, data were processed using a cloud-based pipeline developed at the RGC that uses DNAnexus and AWS to run standard tools for sample-level data production and analysis. Sequence reads were mapped and aligned to the GRCh37/hg19 human genome reference assembly using BWA-mem. Single-nucleotide polymorphisms and INDEL variants and genotypes were called using GATK’s HaplotypeCaller. Standard quality-control filters were applied to called variants. Passing variants were classified, annotated and analyzed using an RGC-implemented Mendelian analysis pipeline to evaluate their potential functional effects. Variants were annotated for their observed frequencies in population control databases such as dbSNP, the 1000 Genomes Project, the Exome Aggregation Consortium Database and internal RGC databases to filter out common polymorphisms and high frequency, likely benign variants. Algorithms for bioinformatic prediction of functional effects of variants (LRT, Poly-phen2, SIFT, CADD and Mutation Taster), along with conservation scores, were incorporated as part of the annotation process of variants and used to inform on the potential deleteriousness of identified candidate variants. Individuals in this study were screened for variants in a list of 28 genes compiled for their reported associations with triglyceride or lipid levels.

Screened genes were *LPL*, *ANGPTL3*, *ANGPTL4*, *ANGPTL8*, *APOA1*, *APOA4*, *APOA5*, *APOC2*, *APOC3*, *APOD*, *COL18A1*, *CREB3L3*, *GALNT2*, *GPIHBP1*, *LMF1*, *PCSK7*, *MLXIPL*, *LIPI*, *USF1*, *ABCA1*, *GPD1*, *GCKR*, *TRIB1*, *BTN2A1*, *LRP8*, *TIMD4*, *ABCG5* and *ABCG8*.

### Imaging

^18^F-FDG-PET/CT and MRI imaging were performed to assess subclinical signs of pancreatic inflammation and injury in study populations. ^18^F-FDG-PET/CT was performed at baseline and after 12 weeks of treatment during the DBTP to examine the impact of treatment on pancreatic inflammation using standardized uptake values, SUV_max_ and SUV_mean_.

MRI was performed at baseline to assess pancreatic injury/inflammation through measurement of apparent diffusion coefficient and levels of hepatic fat fraction. Patients underwent repeat MRI scans at 12 and 24 weeks of treatment to assess changes in pancreatic injury/inflammation and changes in liver hepatic fat fraction.

### Statistical analysis

The primary end point was prespecified as the least squares mean percent reduction in triglycerides from baseline after 12 weeks of evinacumab exposure (combination of DBTP and SBTP) in cohort 3. This was assessed in cohort 3 using asynchronous study periods that were dependent upon treatment group to assess changes in triglycerides after 12 weeks of evinacumab treatment; for patients randomized to evinacumab, the primary analysis included the 12-week DBTP, whereas for patients randomized to placebo the primary analysis included the subsequent 12-week SBTP of active treatment with evinacumab. Point estimates of mean percent changes in triglycerides between the placebo run-in period and each observation were calculated using a mixed-effect model for repeated measures (MMRM) approach.

Before MMRM analysis of the primary end point, fasting serum triglycerides were log-transformed with the aim to provide a normal data distribution. A log-scale s.d. of 0.5 was used based on available evinacumab phase 1 data. To reduce variability of baseline triglyceride measurements, baseline was the mean of the three log-transformed measurements (day –28, day –14 and week 0 (DBTP); weeks 6, 8 and 12 (SBTP)).

The primary end point analysis was based on the percent change in triglycerides from baseline following 12 weeks of treatment with evinacumab in cohort 3. Point estimates of mean percent changes in triglycerides between the placebo run-in period and each observation were calculated using an MMRM approach. The MMRM model assessed within-patient treatment comparisons using an unstructured covariance matrix while accounting for baseline triglyceride values, study visit and baseline triglyceride values by study visit interaction, but not trough levels of evinacumab, which varied and were often below the targeted threshold of 100 mg l^−1^. Study visits were adjusted to the start of evinacumab treatment to pool data from the DBTP and the SBTP. Least squares means with CIs and least squares mean ratios with CIs were used to assess treatment effects.

Secondary end points included the percent triglyceride lowering from baseline following 2–24 weeks repeated i.v. doses of evinacumab; the proportion of patients who achieved at least a 40%, 50%, 60%, 70%, 80% or 90% reduction in triglycerides from baseline and the proportion of patients who achieved a reduction in triglycerides below 500 mg dl^−1^ after 2–24 weeks evinacumab treatment (not reported); the percent change in post-heparin LPL activity from baseline (not reported); changes in patient-reported abdominal and gastrointestinal symptoms, dietary habits and symptom/dietary impact measures, assessed via questionnaires (not reported); the degree of pancreatic injury/inflammation at baseline and change from baseline following 12 weeks of evinacumab treatment (see further details above); the evaluation of evinacumab pharmacokinetics, total ANGPTL3 levels and anti-drug antibodies during the treatment and follow-up periods (not reported); and the incidence and severity of TEAEs, serious adverse events, laboratory abnormalities and other safety variables.

Post hoc analyses were undertaken to evaluate whether (1) mean triglyceride values at baseline and week 12; (2) percent change in mean triglyceride values at week 12; (3) mean log-transformed triglyceride values from baseline and week 12; and (4) change in mean log-transformed triglyceride values from baseline at week 12 achieved normal distribution using the MMRM model. It was essential to determine whether triglyceride values that do not follow a normal distribution are log-normal, as applying MMRM analysis without confirming the data obey a log-normal distribution, could lead to misinterpretation of data. Accordingly, tests of normality, including Anderson–Darling, Cramer–von Mises, Kolmogorov–Smirnov and Shapiro–Wilk, were performed. These tests demonstrated that log-transformed triglyceride values were not normally distributed, thus median percent changes were additionally determined and analyzed.

For the DBTP and SBTP, both percent change and absolute triglyceride values, as well as safety and other efficacy end points, were summarized descriptively. For the DBTP, post hoc nominal *P* values are provided for descriptive purposes. All efficacy analyses were performed in the full analysis population, which consisted of all randomized patients who received study drug. The safety analysis set included all randomized individuals who received at least one dose, or part of a dose, of study drug. Clinical data were analyzed using SAS v.9.4.

### Reporting summary

Further information on research design is available in the Nature Portfolio Reporting Summary linked to this article.

## Online content

Any methods, additional references, Nature Portfolio reporting summaries, source data, extended data, supplementary information, acknowledgements, peer review information; details of author contributions and competing interests; and statements of data and code availability are available at 10.1038/s41591-023-02222-w.

### Supplementary information

Supplementary Information Study personnel, study sites where patients were screened and enrolled, inclusion criteria, exclusion criteria, Supplementary Tables 1–3 and Supplementary Fig. 1.

Reporting Summary

## Figures and Tables

**Extended Data Table 1:**
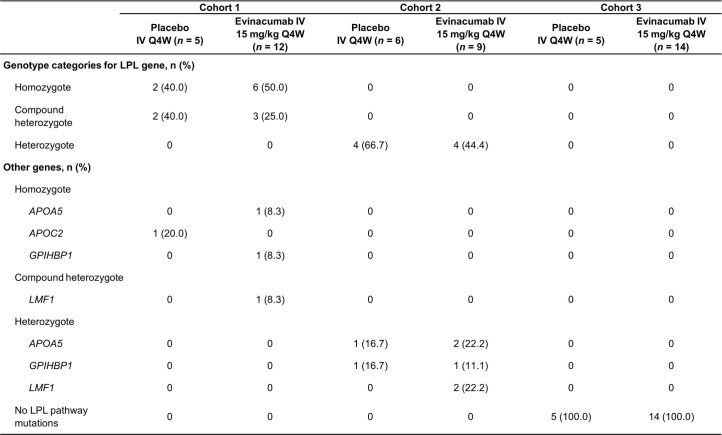
Summary of patients treated in the DBTP by genotype

*APOA5*, gene encoding apolipoprotein A5; *APOC2*, gene encoding apolipoprotein C2; *GPIHBP1*, gene encoding glycosylphosphatidylinositol anchored high-density lipoprotein binding protein 1; IV, intravenous; *LMF1*, gene encoding lipase maturation factor 1; LPL, lipoprotein lipase; Q4W, every 4 weeks.

**Extended Data Table 2:**
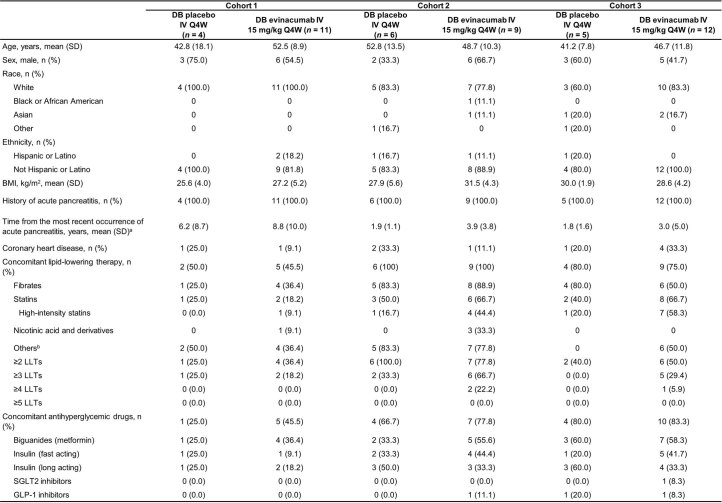
Demographics and baseline characteristics of patients entering the SBTP

^a^Time from diagnosis to study randomization. ^b^Includes omega-3-acid ethyl ester, omega-3 fatty acids, eicosapentaenoic acid ethyl ester, ezetimibe, fish oil, combination of docosahexaenoic acid, eicosapentaenoic acid and fish oil, eicosapentaenoic acid and omega-3 triglycerides. BMI, body mass index; CHD, coronary heart disease; DB, double blind; GLP-1, glucagon-like peptide-1; IV, intravenous; Q4W, every 4 weeks; SD, standard deviation; SGLT2, sodium glucose co-transporter 2

**Extended Data Table 3:**
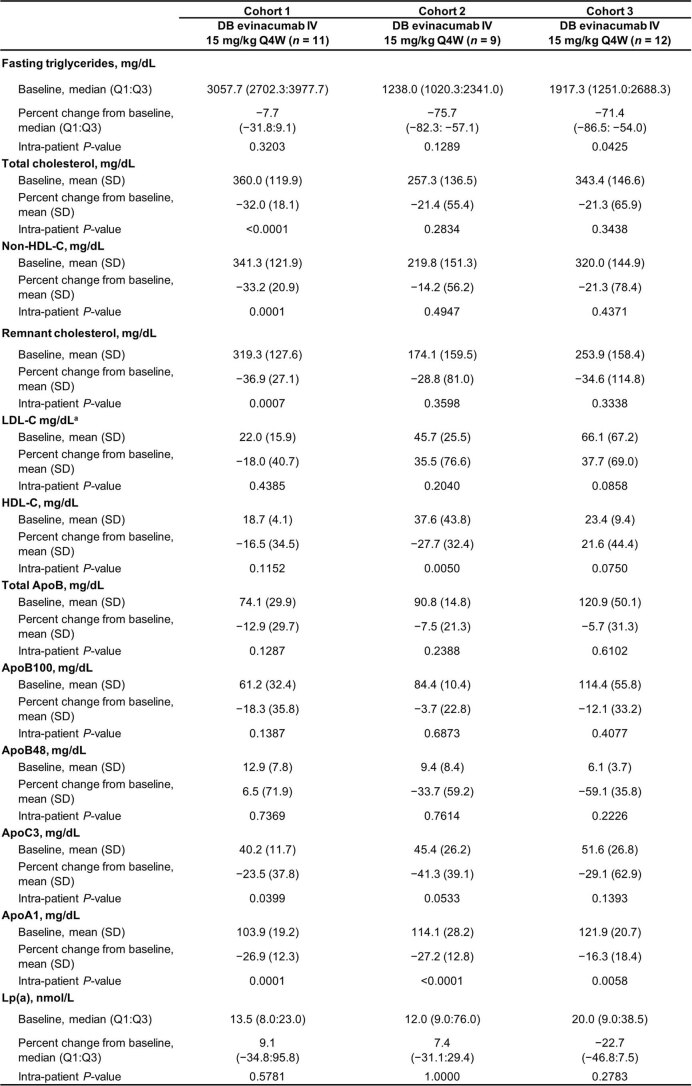
Change in lipid/lipoprotein parameters from baseline to week 24

^a^LDL-C was determined by ultracentrifugation. Apo, apolipoprotein; DB, double blind; HDL-C, high-density lipoprotein choleserol; IV, intravenous; LDL-C, low-density lipoprotein cholesterol; Lp(a), lipoprotein(a); Q1, first quartile; Q3, third quartile; Q4W, every 4 weeks; SD, standard deviation.

**Extended Data Table 4:**
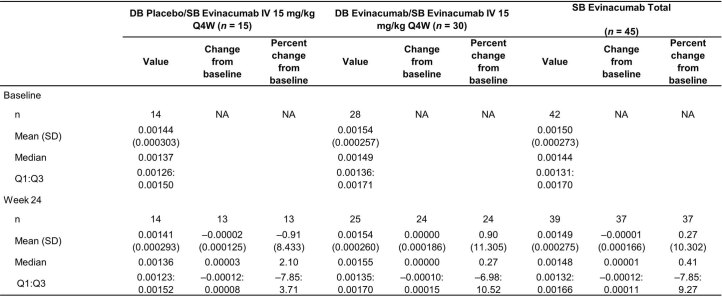
Apparent diffusion coefficient over time – MRI analysis set during the SBTP

DB, double blind; IV, intravenous; MRI, magnetic resonance imaging; Q4W, every 4 weeks; SB, single-blind; SBTP, single-blind treatment period; SD, standard deviation.

**Extended Data Table 5:**
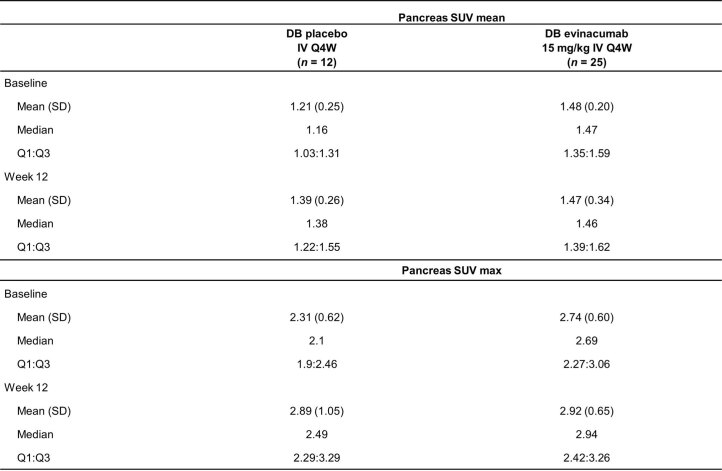
Pancreas SUV mean and SUV max

DB, double blind; IV, intravenous; Q4W, every 4 weeks; SD, standard deviation; SUV, standardized uptake value

**Extended Data Table 6:**
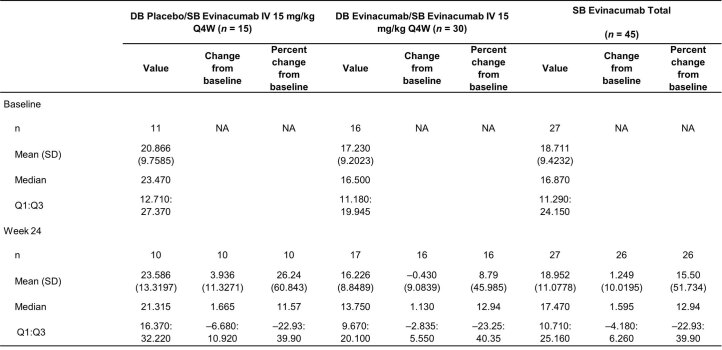
Percent hepatic fat fraction over time during the SBTP

DB, double blind; IV, intravenous; MRI, magnetic resonance imaging; Q4W, every 4 weeks; SB, single-blind; SBTP, single-blind treatment period; SD, standard deviation.

## Data Availability

Qualified researchers may request access to study documents (including the clinical study report, study protocol with any amendments, blank case report form and statistical analysis plan) that support the methods and findings reported in this manuscript. Individual anonymized participant data will be considered for sharing once the product and indication has been approved by major health authorities (for example, FDA, EMA and PMDA), if there is legal authority to share the data and there is not a reasonable likelihood of participant re-identification. Requests should be submitted to https://vivli.org/. GRCh37/hg19 human genome reference assembly can be accessed via https://www.ncbi.nlm.nih.gov/data-hub/genome/GCF_000001405.40/. The following population control databases were utilized: dbSNP, accessed via https://www.ncbi.nlm.nih.gov/snp/; the 1000 Genomes Project, accessed via https://www.internationalgenome.org/; and the Exome Aggregation Consortium Database, accessed via https://gnomad.broadinstitute.org/.
