# Supplementary material for: Evinacumab in severe hypertriglyceridemia with or without lipoprotein lipase pathway mutations: a phase 2 randomized trial
Source: Nat Med. 2023 Mar 6;29(3):729–37. doi: 10.1038/s41591-023-02222-w (PMC10033404; doi:10.1038/s41591-023-02222-w)

nature medicine

Article https://doi.org/10.1038/s41591-023-02222-w

Evinacumab in severe hypertriglyceridemia with or without lipoprotein lipase pathway mutations: a phase 2 randomized trial

In the format provided by the authors and unedited

Study personnel

List of principal investigators per study site where patients were screened and enrolled.

Study center no.	Patients enrolled by site	Investigator (last name, first name)	Professional degree(s)	Institutional affiliation(s)
124002	7	Gaudet, Daniel	MD, PhD, CSPQ	ECOGENE-21, 930 Jacques-Cartier Est, Bureau B-210, Chicoutimi, Quebec, G7H 7K9, Canada
124003	4	Bergeron, Jean	MD	Clinique des Maladies Lipidiques de Quebec, 2600 Boulevard Laurier, Bureau 880, Quebec, G1V 4W2, Canada
380001	4	Rubba, Paolo	MD	Dipartimento di Medicina Clinica e Chirurgia dell' Universita' degli Studi di Napoli Federico II, AO.U. Federico II, Via Sergio Pansini 5, Napoli 80131, Italy
380002	1	Arca, Marcello	MD	Dipartmento di Medicina Traslazionale e di Precisione, Universita degli Studi di Roma "La Sapienza", Viale dell'Universita 37, 00185 Roma. Italy<br>Dipartimento di Medicina Interna e Specialita Mediche, A.O.U. Policlinico Umberto I di Roma Viale del Policlinico 155, Roma 00161, Italy
826001	2	Soran, Handrean	MD, FRCP, MRCP	Manchester University Hospital NHS Foundation Trust, Oxford Road, Manchester, M13 9W, UK
826002	2	Nair, Devaki	MBBS, FRCP	Royal Free London NHS Foundation Trust, Royal Free Hospital, Pond Street, London, NW3 2QG, UK
826003	1	Wierzbicki, Anthony	BMBCh, DPhil, FRCPath	Guy's & St. Thomas NHS Hospitals Foundation Trust, Dept. Chemical Pathology 5th Floor North Wing, St. Thomas Hospital, Westminster Bridge Road, London, SE1 7EH, UK
826004	1	Dawson, Charlotte	MBBS, PhD, MRCP	University Hospitals Birmingham NHS Foundation Trust, New Queen Elizabeth Hospital Birmingham, Mindelsohn Way, Edgbaston, Birmingham, B15 2TH, UK
840001	4	Rader, Daniel J.	MD	University of Pennsylvania, 11-125 SCTR, 3400 Civic Center Blvd, Philadelphia, PA 19104, USA

840002	2	Ahmad, Zahid	MD	UT Southwestern Medical Center, 5323 Harry Hines Boulevard, K5-214, Dallas, TX 75390, USA
840003	4	Kershaw, Erin E.	MD	University of Pittsburgh, 200 Lothrop Street, Pennsylvania, PA BST 10W, USA
840007	3	Moriarty, Patrick M.	MD	University of Kansas Medical Center, Mailstop 3008, 3901 Rainbow Blvd, Kansas City, KS 66160, USA
840008	1	Kidambi, Srividya	MD	Froedtert Health & The Medical College of Wisconsin, 8701 Watertown Plank Road, Milwaukee, WI 53226, USA
840009	3	Ballantyne, Christie M.	MD	Baylor College of Medicine, Center for Cardiometabolic Disease Prevention, 6655 Travis Street, Suite 320, Houston, TX 77030, USA
840010	7	Rosenson, Robert S.	MD, FACP, FACC, FAHA, FNLA, FESC, FCCP	Icahn School of Medicine at Mount Sinai, 1425 Madison Avenue, MC Level, Freedom Trial Office, New York, NY 10029, USA
840014	2	Burkle, Jaime	MD, FACC	Piedmont Atlanta Hospital, 1698 Peachtree Road NW, Atlanta, Georgia, GA 30309, USA
840015	4	Baum, Seth J.	MD, FACC, FACPM, FAHA, FNLA, FASPC	Excel Medical Clinical Trials, L.L.C., 7900 Glades Road, Suite 400, Boca Raton, Florida, FL 33434, USA

Study sites where patients were screened and enrolled

Country	Total number of sites by country	Patients enrolled per country
Canada	2	11
Italy	2	5
UK	4	6
USA	9	30

Inclusion criteria

A patient must meet the following criteria to be eligible for inclusion in the study:

1. Adults aged ≥18 to 75 years at screening.

2. Previous documentation in the patient's medical records of a fasting serum triglyceride measurement ≥1,000 mg/dL (11.3 mmol/L) on more than one occasion, and all fasting triglyceride values ≥500 mg/dL (5.6 mmol/L) at screening.[a]

   [a]Triglycerides will be measured at least twice and at least 4 days apart during the screening period.

3. History of a hospitalization and diagnosis of acute pancreatitis in the past 10 years.[a]

   [a]Sponsor may elect to drop the time specification if sufficient enrollment does not occur within a reasonable period of time.

4. On a stable lipid-modifying diet with or without medications (e.g., statins, niacin, omega-3 fatty acids). Lipid-modifying diet and doses of medications should be stable for at least 4 weeks (6 weeks for fibrates, 8 weeks for proprotein convertase subtilisin/kexin type 9 inhibitors) prior to screening.

5. Body mass index of 18–40 kg/m^2.

6. Willing to consume no more than an average of two standard alcoholic drinks per day and a maximum of 15 standard alcoholic drinks per week for the duration of the study.[a]

[a]A standard alcoholic drink is the equivalent of 355 mL (12 oz) of beer, 148 mL (5 oz) of wine, or 44 mL (1.5 oz) of hard liquor.

7. Willing to refrain from consumption of alcohol for 24 hours prior to each study visit.

8. Willing to refrain from cigarette smoking for 24 hours prior to each study visit.

9. Willing to consistently maintain previously recommended diet and exercise regimen for duration of the study.

10. Willing and able to comply with clinic visits and study-related procedures.

11. Provide signed informed consent.

12. Able to understand and complete study-related questionnaires

Exclusion criteria

A patient who meets any of the following criteria will be excluded from the study:

1. A hospital or clinic discharge diagnosis of acute pancreatitis within 12 weeks of screening.

2. Lipid apheresis or plasma exchange treatment within the last 4 weeks, or plans to undergo apheresis or plasma exchange during the time frame of the study.

3. History of class 3/4 heart failure at any time in the past, or hospitalization for heart failure, diagnosis of a myocardial infarction, stroke, transient ischemic attack, unstable angina, coronary artery bypass graft, percutaneous coronary intervention, or carotid surgery/stenting within 3 months before the screening visit.

4. History of bleeding disorders, esophageal varices, heparin-induced thrombocytopenia, or contraindications to receiving heparin (e.g., allergic reaction).

5. New clinically significant findings on 12-lead electrocardiogram that would place the patient at risk or interfere with participation in the study.

6. Dose(s) of any permitted concomitant medications that have changed in the time period prior to screening.

7. Presence of any clinically significant uncontrolled endocrine disease known to influence serum lipids or lipoproteins.[a]

   [a]Patients on thyroid replacement therapy are eligible if the dosage of thyroxine has been stable for at least 12 weeks prior to screening visit.

8. Use of systemic corticosteroids, unless used as replacement therapy for pituitary/adrenal disease (in which case patient must be on a stable regimen for at least 6 weeks prior to the screening visit).[a]

   [a]Topical, intra-articular, nasal, inhaled, and ophthalmic steroid therapies are not considered as systemic and are allowed.

9. Calculated estimated glomerular filtration rate <45 mL/min (according to 4-variable Modification of Diet in Renal Disease study equation).

10. History of drug or alcohol abuse (including binge drinking which typically involves consumption of approximately four drinks for women and five drinks for men in a few hours) within 1 year of screening.

11. Exposure to another investigational drug or therapy within 30 days or within at least five half-lives (whichever is longer) prior to the screening visit.

12. Blood donation of any volume within 1 month prior to administration of study drug.

13. Known hypersensitivity to monoclonal antibody therapeutics. Known sensitivity to any of the components of the investigational product formulation.

14. History of malignancy within 5 years prior to screening (other than successfully treated non-metastatic cutaneous squamous cell or basal cell carcinoma and/or localized carcinoma in situ of the cervix).

15. Any medical or psychiatric condition that, in the opinion of the investigator, would place the patient at risk, interfere with participation in the study, or interfere with the interpretation of study results (e.g., cirrhosis or chronic active hepatitis, nephrotic syndrome, uncontrolled diabetes, uncontrolled hypertension).

16. Previous treatment with Glybera® in the past 5 years or treatment with lomitapide or mipomersen in the past 6 months.

17. Positive serum human chorionic gonadotrophin pregnancy test at the screening visit.

18. Creatine phosphokinase ≥3 x upper limit of normal (ULN) at the screening visit.

19. Aspartate aminotransferase or alanine aminotransferase ≥3 x ULN.

20. Thyroid-stimulating hormone >1.5 x ULN or < lower limit of normal

21. Platelet count <75,000.

22. Any patient who is the investigator or any sub-investigator, research assistant, study coordinator, or other staff directly involved in the conduct of the protocol, or a family member of staff involved in the conduct of the protocol.

23. Pregnant or breastfeeding women.

24. Women of childbearing potential[a] who are unwilling to practice a highly effective birth control method prior to the initial dose, during the study, and for 24 weeks after the last administration of study drug. Highly effective contraception measures include:
    - Stable use of combined (estrogen- and progestogen-containing) hormonal contraception associated with inhibition of ovulation initiated two or more menstrual cycles prior to screening:
        - Oral
        - Intravaginal
        - Transdermal
    - Stable use of progestogen-only hormonal contraception associated with inhibition of ovulation initiated two or more menstrual cycles prior to screening:
        - Oral
        - Injectable
        - Implantable
    - Intrauterine device
    - Intrauterine hormone-releasing system

- Bilateral tubal ligation.
- Vasectomized partner.

  Note: Vasectomized partner is a highly effective birth control method provided that the partner is the sole sexual partner of the study participant and that the vasectomized partner has received medical assessment of the surgical success.
- Sexual abstinence.

  Note: Sexual abstinence is considered a highly effective method only if defined as refraining from heterosexual intercourse during the entire period of risk associated with study treatments.

[a]Postmenopausal women must be amenorrhoeic for at least 12 months in order not to be considered of childbearing potential. Postmenopausal status will be confirmed by measurement of follicle-stimulating hormone. Pregnancy testing and contraception are not required for women with documented hysterectomy and/or oophorectomy. Oocyte donation is prohibited during the study and for 24 weeks after the last administration of study drug.

25. Men who are sexually active with women of childbearing potential and are unwilling to consistently use condoms during the study drug treatment period and for 24 weeks after the last injection of study drug, regardless of vasectomy status. Sperm donation is prohibited during the study and for up to 24 weeks after the last injection of study drug.

Supplementary Table 1. Genetic variants identified in LPL pathway- or hypertriglyceridemia-associated genes

Patient ID	Gene	Gene name	Nucleotide change	Amino acid change	Zygosity	Predicted effect	Genotypes	Baseline fasting triglycerides, mg/dL
**Cohort 1**								
1	*LPL*	*Lipoprotein lipase*	c.C701T	p.P234L[a]	Homozygous	Deleterious	HET=77/HOM=19	3978
2	*LPL*	*Lipoprotein lipase*	c.C701T	p.P234L[a]	Homozygous	Deleterious	HET=77/HOM=19	2267
3	*LPL*	*Lipoprotein lipase*	c.G644A	p.G215E[a]	Homozygous	Deleterious	HET=115/HOM=0	3122
4	*LPL*	*Lipoprotein lipase*	c.G644A	p.G215E[a]	Homozygous	Deleterious	HET=115/HOM=0	4095
5	*LPL*	*Lipoprotein lipase*	c.T755C	p.I252T[a]	Homozygous	Deleterious	HET=19/HOM=0	3058
6	*LPL*	*Lipoprotein lipase*	c.G844T	p.E282X	Homozygous	Deleterious	HET=0/HOM=0	2702
7	*LPL*	*Lipoprotein lipase*	c.C621G	p.D207E[a]	Homozygous	Deleterious	HET=0/HOM=0	3918
8	*GPIHBP1*	*Glycosylphosphatidylinositol anchored high density lipoprotein binding protein 1*	c.G230A	p.C77Y	Homozygous	Deleterious	HET=0/HOM=0	3864
9	*LPL*	*Lipoprotein lipase*	c.G106A	p.D36N[b]	Heterozygous	Neutral	HET=5382/HOM=67	3224
	LMF1	*Lipase maturation factor 1*	c.G1078C	p.G360R	Heterozygous	Deleterious	HET=1/HOM=0	
	LMF1	*Lipase maturation factor 1*	c.C441G	p.N147K	Heterozygous	Deleterious	HET=22/HOM=0	
	LMF1	*Lipase maturation factor 1*	c.G1052A	p.R351Q	Heterozygous	Tolerated	HET=564/HOM=2	
10	*LPL*	*Lipoprotein lipase*	c.C701T	p.P234L[a]	Homozygous	Deleterious	HET=77/HOM=19	4175
11	*LPL*	*Lipoprotein lipase*	c.G644A	p.G215E[a]	Heterozygous	Deleterious	HET=115/HOM=0	2960
	LPL	*Lipoprotein lipase*	c.C701T	p.P234L[a]	Heterozygous	Deleterious	HET=77/HOM=19	
	LMF1	*Lipase maturation factor 1*	c.C1060T	p.R354W	Heterozygous	Deleterious	HET=5393/HOM=50	
12	*LPL*	*Lipoprotein lipase*	c.G644A	p.G215E[a]	Heterozygous	Deleterious	HET=115/HOM=0	3806
	APOA5	*Apolipoprotein A5*	c.1019-3C>A	Splicing	Heterozygous	Deleterious	HET=1/HOM=0	
	APOA5	*Apolipoprotein A5*	c.161+3G>A	Splicing	Heterozygous	Deleterious	HET=30/HOM=0	
13	*LPL*	*Lipoprotein lipase*	c.C701T	p.P234L[a]	Heterozygous	Deleterious	HET=77/HOM=19	4886
	LPL	*Lipoprotein lipase*	c.G106A	p.D36N[b]	Heterozygous	Neutral	HET=5382/HOM=67	
14	*LPL*	*Lipoprotein lipase*	c.C701T	p.P234L[a]	Homozygous	Deleterious	HET=77/HOM=19	2724
15	*LPL*	*Lipoprotein lipase*	c.C701T	p.P234L[a]	Heterozygous	Deleterious	HET=77/HOM=19	2581

	LPL	*Lipoprotein lipase*	c.G106A	p.D36N[b]	Heterozygous	Neutral	HET=5382/HOM=67	
16	*APOC2*	*Apolipoprotein C*	c.G215C	p.R72T	Homozygous	Deleterious	HET=1/HOM=1	3931
17	*APOA5*	*Apolipoprotein A5*	c.49+5G>A	Splicing	Homozygous			804
**Cohort 2**								
18	*APOA5*	*Apolipoprotein A5*	c.C883T	p.Q295X	Heterozygous	Deleterious	HET=2/HOM=0	1737
	ANGTPL8	*Angiopoietin like 8*	c.C361T	p.Q121X	Heterozygous	Deleterious	HET=417/HOM=0	
19	*LPL*	*Lipoprotein lipase*	c.G644A	p.G215E[a]	Heterozygous	Deleterious	HET=115/HOM=0	4324
20	*LPL*	*Lipoprotein lipase*	c.58_71del insCCTC	p.A20Pfs*21	Heterozygous	Deleterious	HET=0/HOM=0	966
21	*APOA5*	*Apolipoprotein A5*	c.C56G	p.S19W[b]	Heterozygous	Deleterious	HET=22152/HOM=1130	3704
22	*LPL*	*Lipoprotein lipase*	c.C701T	p.P234L[a]	Heterozygous	Deleterious	HET=77/HOM=19	2341
23	*LPL*	*Lipoprotein lipase*	c.G644A	p.G215E[a]	Heterozygous	Deleterious	HET=115/HOM=0	1044
24	*LPL*	*Lipoprotein lipase*	c.A953G	p.N318S	Heterozygous	Tolerated	HET=5276/HOM=58	921
	LMF1	*Lipase maturation factor 1*	c.C1351T	p.R451W	Heterozygous	Deleterious	HET=998/HOM=5	
25	*LPL*	*Lipoprotein lipase*	c.G644A	p.G215E[a]	Heterozygous	Deleterious	HET=115/HOM=0	4010
	LRP8	*LDL receptor related protein 8*	c.T71G	p.L24R	Heterozygous	Benign	HET=7069/HOM=0	
	LRP8	*LDL receptor related protein 8*	c.74_76del	p.25_26del	Heterozygous	NA	HET=8/HOM=0	
26	*APOA5*	*Apolipoprotein A5*	c.161+5G>C	Splicing	Heterozygous	Deleterious	HET=9/HOM=0	1238
	COL18A1	*Collagen type XVIII alpha 1 chain*	c.G3181A	p.V1061M	Heterozygous	Deleterious	HET=39/HOM=0	
27	*LPL*	*Lipoprotein lipase*	c.91_95del	p.R32Ffs*7	Heterozygous	Deleterious	HET=2/HOM=0	1546
	COL18A1	*Collagen type XVIII alpha 1 chain*	c.T4772C	p.M1591T	Heterozygous	Deleterious	HET=1/HOM=0	
	BTN2A1	*Butyrophilin subfamily 2 member A1*	c.G857A	p.R286Q	Heterozygous	Tolerated	HET=30/HOM=0	
	GPD1	*Glycerol-3-phosphate dehydrogenase 1*	c.A160G	p.I54V	Heterozygous	Tolerated	HET=1033/HOM=13	
28	*LPL*	*Lipoprotein lipase*	c.G106A	p.D36N	Heterozygous	Deleterious	HET=5382/HOM=67	379
	GPD1	*Glycerol-3-phosphate dehydrogenase 1*	c.G347A	p.R116H	Heterozygous	Tolerated	HET=13/HOM=0	
29	*LPL*	*Lipoprotein lipase*	c.G644A	p.G215E[a]	Heterozygous	Deleterious	HET=115/HOM=0	769
	ABCG8	*ATP binding cassette subfamily G member 8*	c.A1225G	p.N409D	Heterozygous	Deleterious	HET=224/HOM=1	
30	*LPL*	*Lipoprotein lipase*	c.C701T	p.P234L[a]	Heterozygous	Deleterious	HET=77/HOM=19	1020

	GPD1	*Glycerol-3-phosphate dehydrogenase 1*	c.A160G	p.I54V	Heterozygous	Deleterious	HET=1033/HOM=13	
	LIPI	*Lipase I*	c.T856C	p.C286R	Heterozygous	Deleterious	HET=2880/HOM=21	
	LMF1	*Lipase maturation factor 1*	c.C1060T	p.R354W	Heterozygous	Tolerated	HET=5393/HOM=50	
31	*LPL*	*Lipoprotein lipase*	c.C701T	p.P234L[a]	Heterozygous	Deleterious	HET=77/HOM=19	809
	APOB	*Apolipoprotein B*	c.G4265A	p.C1422Y	Heterozygous	Neutral	HET=355/HOM=0	
32	*LMF1*	*Lipase maturation factor 1*	c.T713C	p.M238T	Heterozygous	Deleterious	HET=1/HOM=0	4942
**Cohort 3**								
33				No findings in LPL pathway genes				1108
34				No findings in LPL pathway genes				1031
35				No findings in LPL pathway genes				1306
36				No findings in LPL pathway genes				2769
37				No findings in LPL pathway genes				2607
38				No findings in LPL pathway genes				942
39	*MLXIPL*	*MLX interacting protein like*	c.A1372C	p.T458P	Heterozygous	Deleterious	HET=252/HOM=0	1549
40	*LMF1*	*Lipase maturation factor 1*	c.C1060T	p.R354W	Heterozygous	Deleterious	HET=5393//HOM=50	1923
	LRP8	*LDL receptor related protein 8*	c.T71G	p.L24R	Heterozygous	Benign	HET=7069/HOM=0	
41				No findings in LPL pathway genes				2405
42				No findings in LPL pathway genes				1196
43				No findings in LPL pathway genes				205
44	*ANGPTL4*	*Angiopoietin like 4*	c.G499A	p.E167K	Heterozygous	Tolerated	HET=94/HOM=0	3740
45	*ABCG1*	*ATP binding cassette subfamily G member 1*	c.G1006A	p.G336R	Heterozygous	Tolerated	HET=170/HOM=1	1912
	BTN2A1	*Butyrophilin subfamily 2 member A1*	c.T1460C	p.F487S	Heterozygous	Deleterious	HET=0/HOM=0	
	COL18A1	*Collagen type XVIII alpha 1 chain*	c.T1181C	p.V394A	Heterozygous	Tolerated	HET=98/HOM=0	
56				*No findings in LPL pathway genes*				401
47	*COL18A1*	*Collagen type XVIII alpha 1 chain*	c.C3842G	p.P1281R	Heterozygous	Deleterious	HET=4666	8310
				No findings in LPL pathway genes				
48	*LPL*	*Lipoprotein lipase*	c.A953G	p.N318S	Heterozygous	Tolerated	HET=5276/HOM=58	1746
	COL18A1	*Collagen type XVIII alpha 1 chain*	c.C56T	p.A19V	Heterozygous	Deleterious	HET=452/HOM=3	

	COL18A1	*Collagen type XVIII alpha 1 chain*	c.G3664A	p.V1222M	Heterozygous	Tolerated	HET=601/HOM=6	
49			No findings in LPL pathway genes					1022
50	*LPL*	*Lipoprotein lipase*	c.A953G	p.N318S	Heterozygous	Tolerated	HET=5276/HOM=58	1496
	ABCG8	*ATP binding cassette subfamily G member 8*	c.G712A	p.E238K	Heterozygous	Deleterious	HET=423/HOM=0	
	ABCA1	*ATP binding cassette subfamily A member 1*	c.T2903C	p.M968T	Heterozygous	Deleterious	HET=29/HOM=0	
	ABCG5	*ATP binding cassette subfamily G member 5*	c.A1864G	p.M622V	Heterozygous	Tolerated	HET=2076/HOM=11	
	COL18A1	*Collagen type XVIII alpha 1 chain*	c.C560T	p.T187I	Heterozygous	Tolerated	HET=2706/HOM=18	
51			No findings in LPL pathway genes					1974

[a]Previously reported pathogenic variant. [b]Variant considered a risk factor but not causative.

Supplementary Table 2. Summary of TEAEs in any treatment group during the SBTP

No. of Patients, *n* (%)	DB placebo IV Q4W (*n* = 15)	DB evinacumab 15 mg/kg IV Q4W (*n* = 32)
Patients with at least one TEAE	13 (86.7)	25 (78.1)
Patients with at least one serious TEAE	4 (26.7)	11 (34.4)
Patients with at least one TEAE resulting in discontinuation of treatment	0	0
Patients with any TEAE resulting in death	0	0
TEAEs occurring in >5% of patients in any group		
Acute pancreatitis	4 (26.7)	8 (25.0)
Abdominal pain	2 (13.3)	4 (12.5)
Urinary tract infection	1 (6.7)	4 (12.5)
Abdominal pain (upper)	1 (6.7)	3 (9.4)
Nasopharyngitis	1 (6.7)	3 (9.4)
Abdominal discomfort	0	2 (6.3)
Blood magnesium decreased	0	2 (6.3)
Bronchitis	1 (6.7)	2 (6.3)
Fatigue	1 (6.7)	2 (6.3)
Headache	2 (13.3)	2 (6.3)
Hypertension	0	2 (6.3)
Muscle spasms	0	2 (6.3)
Vomiting	1 (6.7)	2 (6.3)
Acute kidney injury	1 (6.7)	1 (3.1)
Clostridium difficile infection	1 (6.7)	1 (3.1)
Diabetes mellitus	2 (13.3)	1 (3.1)
Food poisoning	1 (6.7)	1 (3.1)
Influenza	1 (6.7)	1 (3.1)
Influenza-like illness	1 (6.7)	1 (3.1)
Metabolic acidosis	1 (6.7)	1 (3.1)
Nausea	2 (13.3)	1 (3.1)
Oropharyngeal pain	1 (6.7)	1 (3.1)
Upper respiratory tract infection	1 (6.7)	1 (3.1)

Alanine aminotransferase increased	2 (13.3)	0
Aspartate aminotransferase increased	2 (13.3)	0
Diarrhea	2 (13.3)	0
Acute respiratory failure	1 (6.7)	0
Abdominal distension	1 (6.7)	0
Anemia	1 (6.7)	0
Atelactasis	1 (6.7)	0
Azotemia	1 (6.7)	0
Blood lactate dehydrogenase increased	1 (6.7)	0
Bursitis	1 (6.7)	0
Delirium	1 (6.7)	0
Diverticulum	1 (6.7)	0
Dyspnea	1 (6.7)	0
Enteritis	1 (6.7)	0
Eructation	1 (6.7)	0
Esophagitis	1 (6.7)	0
Flatulence	1 (6.7)	0
Gastritis	1 (6.7)	0
Herpes zoster	1 (6.7)	0
Hiccups	1 (6.7)	0
Hypocalcemia	1 (6.7)	0
Ileus	1 (6.7)	0
Infusion-related infection	1 (6.7)	0
Laryngitis	1 (6.7)	0
Melanocytic nevus	1 (6.7)	0
Migraine	1 (6.7)	0
Nasal congestion	1 (6.7)	0
Nodal osteoarthiritis	1 (6.7)	0
Oral candidiasis	1 (6.7)	0
Pancreatic failure	1 (6.7)	0
Renal cyst	1 (6.7)	0
Renal impairment	1 (6.7)	0
Sciatica	1 (6.7)	0
Sinusitis	1 (6.7)	0

Type 2 diabetes mellitus	1 (6.7)	0

DB, double blind; IV, intravenous; Q4W, every 4 weeks; SBTP, single-blind treatment period; TEAE, treatment-emergent adverse event.

Supplementary Table 3. Acute pancreatitis events during the DBTP and SBTP

Age/sex/cohort	Study day of AP event	Study day of last evinacumab dose prior to AP onset and interval to AP event (days)	Proximate study visit TG level (mg/dL) prior to event (days prior to AP event)	Proximate evinacumab trough concentration (mg/L) and study day	Local laboratory results at time of AP event	Imaging results at time of AP event
**DBTP**						
65/M/Cohort 1	54	N/A (placebo treatment group)	2,985 (day 42)	N/A (placebo treatment group)	TGs: 1,763 mg/dL (ULN: 168 mg/dL)<br>Serum lipase: 1,909 IU/L (ULN: 82 IU/L)	MRI scan showed signs of acute pancreatitis
29/F/Cohort 3	65	N/A (placebo treatment group)	4,958 (day 51)	N/A (placebo treatment group)	TGs: 1,560 mg/dL (ULN: 150 mg/dL)<br>Serum lipase values not provided	None provided
52/M/Cohort 1	86	57 (29)	4,293 (day 57)	76.8 (trough, day 57)<br>454 (post-infusion day 57)	TGs: 1,006 mg/dL (ULN: 168 mg/dL)<br>Serum lipase: 80 IU/L (ULN: 300 IU/L)	No imaging performed
40/M/Cohort 3	3	1 (2)	2,449 (day 1)	BLQ (day 1 pre-dose)<br>Day 1 post-infusion level not collected	Serum lipase: 369 IU/L (ULN: 118 IU/L)	CT scan showed signs of AP

31/M/Cohort 3	13	1 (12)	2,613 (day 1)	BLQ (day 1 pre-dose)<br>1,320 (day 1 post-infusion)	TGs: 1,178 mg/dL (ULN: 151 mg/dL)<br>Serum lipase: 49 IU/L (ULN: 78 IU/L)	MRI/ultrasound scan did not show signs of AP
**SBTP and off-drug follow-up period**						
45/M/Cohort 3	199	137 (62)	1,775 (day 172)	0.401 (day 199)	Serum lipase: 545 IU/L (ULN: 82 IU/L)	CT scan showed signs of severe pancreatitis
48/M/Cohort 3	240	141 (99)	2,106 (day 197)	112 (day 197)	TGs: >1,575 mg/dL (ULN: 150 mg/dL)<br>Lipase: >6,000 U/L (ULN: 300 U/L)	CT scan showed signs of pancreatitis
52/F/Cohort 2	179	142 (37)	663 (day 166)	103 (day 166)	TGs: 494 mg/dL (obtained 12 days after onset of symptoms; ULN: 199 mg/dL)<br>Lipase: 3,064 U/L (ULN: 393 U/L)	CT showed signs of AP
	271	142 (129)	663 (day 166)	103 (day 166)	TGs: 7,757 mg/dL (ULN: 150 mg/dL)<br>Lipase: 213 U/L (ULN: 70 U/L)	CT showed signs of AP
31/M/Cohort 2	185	141 (44)	7,639 (day 174)	19.4 (day 174)	TGs: >5,680 mg/dL<br>Lipase: 278 U/L (ULN: 82 U/L)	CT showed signs of AP

54/M/Cohort 3	224	141 (83)	201 (day 204)	216 (day 204)	TGs: By report, TGs >1,000 mg/dL at out-of-state hospital while traveling. Upon return home, documented value of 455 mg/dL (obtained 12 days after the onset of symptoms; ULN: 150 mg/dL)	CT obtained 12 days after the onset of symptoms did not show signs of AP
48/F/Cohort 3	219[a]	138 (81)	1,348 (day 194)	17.5 (day 194)	Amylase: 49 U/L<br>Lipase: 38 U/L<br>(no ref. ranges provided)	CT showed signs of AP
	261	138 (123)	6,297 (day 250)	0.145 (day 250)	By report, >8,000 mg/dL on event day and an inpatient level 1,992 mg/dL on hospital day 8<br>Lipase 41 U/L on presentation	CT showed signs of AP
60/F/Cohort 1	183	138 (45)	3,625 (day 169)	185 (day 169)	None provided	By report, no imaging performed
	245	138 (107)	3,162 (day 197)	86.6 (day 197)	By report: TGs: >4,000 mg/dL (ULN: 149 mg/dL)<br>Lipase: Within normal limits (ULN: 71 U/L)	CT performed 1 day after presentation, “with no acute findings and no evidence of pancreatitis”.

58/F/Cohort 2	91	85 (6)	4,990 (day 85)	BLQ (assigned to placebo through day 85 [week 12 of the DBTP])<br>Post-infusion level at day 85 = 678	TG, lipase, and amylase values not provided.	CT performed 2 days after presentation, “which did not show inflammation”
34/F/Cohort 2	112	85 (27)	476 (day 85)	BLQ (assigned to placebo through day 85 [week 12 of the DBTP])<br>Post-infusion level at day 85 = 449	None provided	None provided
59/F/Cohort 1	93	84 (9)	2,011 (day 84)	106 (trough, day 84)<br>784 (post-infusion day 84)	TGs: 1,753 mg/dL (ULN: 168 mg/dL)<br>Lipase: 313 U/L (ULN: 82 U/L)	MRI showed signs of AP
	141	113 (28)	3,026 (day 113)	No PK measurement at day 113<br>Trough at day 146 was 113 mg/L	TGs: 489 mg/dL (ULN: 168 mg/dL)<br>By report, diagnosis was “pancreatitis without elevated enzymes”, but no values were provided.	CT scan showed reduced inflammation compared to previous MRI; “no significant findings”
	261	146 (115)	1,752 (day 252)	0.267 (day 252)	TGs: 4,185 mg/dL (ULN: 168 mg/dL)	No pancreatic imaging studies reported

27/F/Cohort 3	92	85 (7)	7,406 (day 85)	0.895 mg/L (trough, day 85)<br>360 mg/L (post-infusion day 85)	TGs: 1,588 mg/dL at presentation, increasing to 5,099 mg/dL during hospitalization (ULN not provided)<br>Lipase: 392 U/L (ULN: 78 U/L)	CT showed signs of AP
	168	141 (27)	7,814 (day 141)	27.1 mg/L (trough, day 141)<br>279 mg/L (post-infusion, day 141)	TGs: 5,063 mg/dL<br>Lipase: 219 U/L (ULN: 82 U/L)	CT showed signs of AP
	296	141 (155)	2,557 (day 211)	0.267 (day 211)	TGs: 5,680 mg/dL (ULN: 500 mg/dL)<br>Lipase: 202 U/L (ULN: 78 U/L)	CT showed signs of AP
31/M/Cohort 3	140	107 (33)	2,563 (day 140)	14.2 (trough, day 140)	TGs: 1,741 mg/dL (ULN: 150 mg/dL)<br>Lipase: 70 (no units or ULN provided)	No imaging reported
	213	140 (73)	2,113 (day 170)	19.3 (day 170)	TGs: >5,680 mg/dL (ULN: 150 mg/dL)<br>Amylase/lipase: not reported	No imaging reported

[a]Event was not recorded in the clinical database as an AP event, but was considered as such by the sponsor based on further information.

AP, acute pancreatitis; BLQ, below the limit of quantification; CT, computed tomography; MRI, magnetic resonance imaging; TG, triglyceride; ULN, upper limit of normal.

Supplementary Figure 1. Concentration of total evinacumab and triglycerides over time in individual patients who reported an episode of acute pancreatitis (indicated by black vertical line)

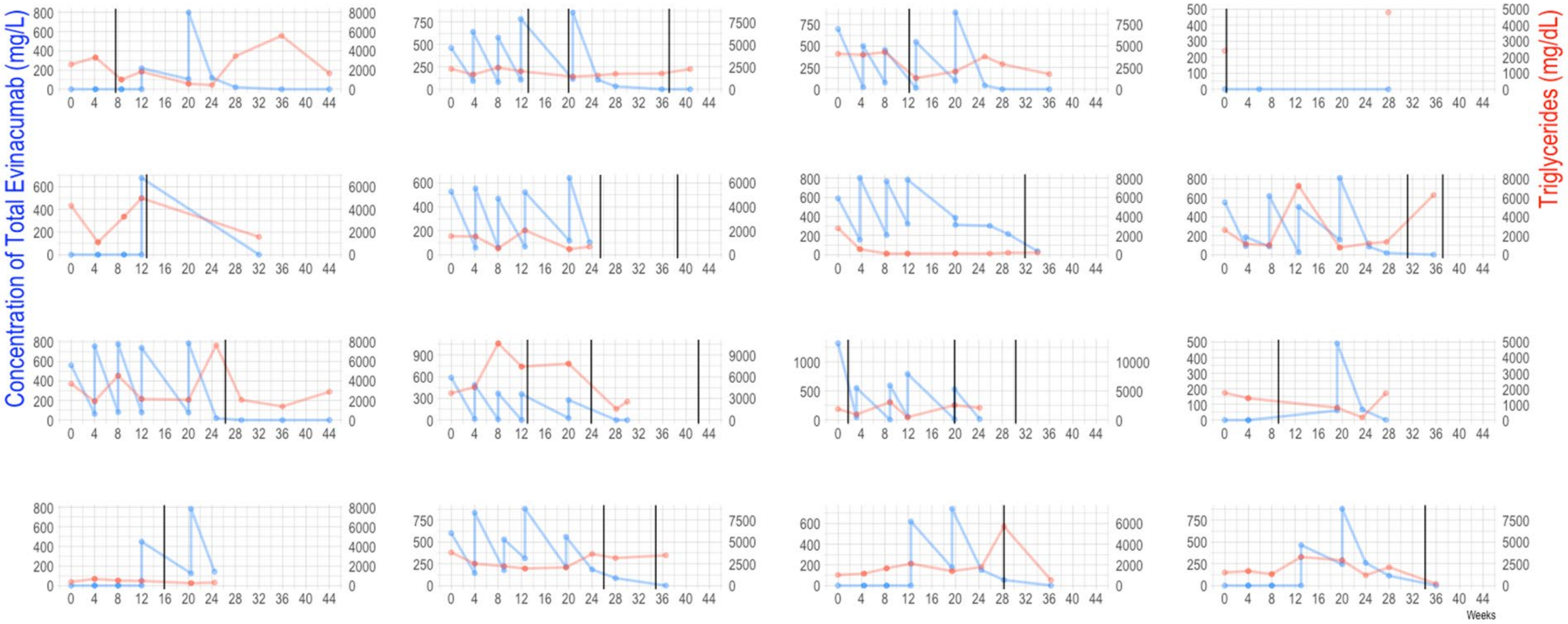

Supplement: Supplementary file 1 — Study personnel, study sites where patients were screened and enrolled, inclusion criteria, exclusion criteria, Supplementary Tables 1–3 and Supplementary Fig. 1. [file 41591_2023_2222_MOESM1_ESM.pdf]
