# Supplementary material for: Evinacumab in severe hypertriglyceridemia with or without lipoprotein lipase pathway mutations: a phase 2 randomized trial
Source: Nat Med. 2023 Mar 6;29(3):729–37. doi: 10.1038/s41591-023-02222-w (PMC10033404; doi:10.1038/s41591-023-02222-w)
Supplement: Supplementary file 2 — Reporting Summary [file 41591_2023_2222_MOESM2_ESM.pdf]

Corresponding author(s): Dr Robert S Rosenson

Last updated by author(s): Dec 14, 2022

Reporting Summary

Nature Portfolio wishes to improve the reproducibility of the work that we publish. This form provides structure for consistency and transparency in reporting. For further information on Nature Portfolio policies, see our Editorial Policies and the Editorial Policy Checklist.

Statistics

For all statistical analyses, confirm that the following items are present in the figure legend, table legend, main text, or Methods section.

n/a	Confirmed	
☐	☒	The exact sample size (*n*) for each experimental group/condition, given as a discrete number and unit of measurement
☒	☐	A statement on whether measurements were taken from distinct samples or whether the same sample was measured repeatedly
☐	☒	The statistical test(s) used AND whether they are one- or two-sided<br>*Only common tests should be described solely by name; describe more complex techniques in the Methods section.*
☒	☐	A description of all covariates tested
☐	☒	A description of any assumptions or corrections, such as tests of normality and adjustment for multiple comparisons
☐	☒	A full description of the statistical parameters including central tendency (e.g. means) or other basic estimates (e.g. regression coefficient) AND variation (e.g. standard deviation) or associated estimates of uncertainty (e.g. confidence intervals)
☐	☒	For null hypothesis testing, the test statistic (e.g. *F*, *t*, *r*) with confidence intervals, effect sizes, degrees of freedom and *P* value noted<br>*Give P values as exact values whenever suitable.*
☒	☐	For Bayesian analysis, information on the choice of priors and Markov chain Monte Carlo settings
☒	☐	For hierarchical and complex designs, identification of the appropriate level for tests and full reporting of outcomes
☒	☐	Estimates of effect sizes (e.g. Cohen's *d*, Pearson's *r*), indicating how they were calculated

Our web collection on statistics for biologists contains articles on many of the points above.

Software and code

Policy information about availability of computer code

Data collection	Not applicable
Data analysis	Clinical data were analyzed using SAS 9.4. Genomic sequence reads were mapped and aligned using BWA-mem. SNP and INDEL variants and genotypes were called using GATK's HaplotypeCaller.

For manuscripts utilizing custom algorithms or software that are central to the research but not yet described in published literature, software must be made available to editors and reviewers. We strongly encourage code deposition in a community repository (e.g. GitHub). See the Nature Portfolio guidelines for submitting code & software for further information.

Data

Policy information about availability of data

All manuscripts must include a data availability statement. This statement should provide the following information, where applicable:

- Accession codes, unique identifiers, or web links for publicly available datasets
- A description of any restrictions on data availability
- For clinical datasets or third party data, please ensure that the statement adheres to our policy

Qualified researchers may request access to study documents (including the clinical study report, study protocol with any amendments, blank case report form, and statistical analysis plan) that support the methods and findings reported in this manuscript. Individual anonymized participant data will be considered for sharing once the product and indication has been approved by major health authorities (e.g., FDA, EMA, PMDA, etc.), if there is legal authority to share the data and there is

not a reasonable likelihood of participant re-identification. Submit requests to https://vivli.org/.
GRCh37/hg19 human genome reference assembly can be accessed via https://www.ncbi.nlm.nih.gov/data-hub/genome/GCF_000001405.40/
The following population control databases were utilized: dbSNP, accessed via https://www.ncbi.nlm.nih.gov/snp/; the 1000 Genomes Project, accessed via https://www.internationalgenome.org/; and the Exome Aggregation Consortium Database, accessed via https://gnomad.broadinstitute.org/.

Human research participants

Policy information about studies involving human research participants and Sex and Gender in Research.

Reporting on sex and gender	The findings are applicable to all sexes and genders.<br>Sex and/or gender was not considered in the study design. The sex of participants was self-reported, and was summarized as part of the baseline characteristic information collected for the trial. Overall, the randomized population who received treatment included 27 (52.9%) males and 24 (47.1%) females.<br><br>No sex- or gender-based analyses have been performed as these were not pre-specified in the study protocol/statistical analysis plan for this trial. Post-hoc sex- or gender-based analyses have not been performed due to the small sample sizes within treatment groups.
Population characteristics	Male or female adults aged 18 to 75 years with severe severe hypertriglyceridemia (fasting serum triglycerides >500 mg/dL at screening on two separate occasions; documented medical history of fasting triglycerides ≥1000 mg/dL) with a history of hospitalization for acute pancreatitis were enrolled based on genotype according to the presence of LOF mutations in LPL pathway genes.
Recruitment	Patients were recruited at 17 study sites across four countries (Canada, Italy United Kingdom, and United States). Efforts were made to select sites with a large population of well-characterized patients who have previously undergone gene sequencing and other procedures to understand the etiology of their hypertriglyceridemia. All participants were assessed for eligibility based on predefined study inclusion/exclusion criteria, as assessed during the screening visit. The small number of patients recruited and the variability in their serum triglycerides are a limitation of this trial and may influence the interpretation of the results.
Ethics oversight	Quorum Review, Comitato Etico dell Universita, Policlinico Umberto I di Roma, North West – Greater Manchester South Research Ethics Committee, The University of Pennsylvania Institutional Review Board, The University of Texas Institutional Review Board, Western IRB, Human Research Protection Program The University of Kansas Medical Center, Copernicus IRB

Note that full information on the approval of the study protocol must also be provided in the manuscript.

Field-specific reporting

Please select the one below that is the best fit for your research. If you are not sure, read the appropriate sections before making your selection.

☒ Life sciences ☐ Behavioural & social sciences ☐ Ecological, evolutionary & environmental sciences

For a reference copy of the document with all sections, see nature.com/documents/nr-reporting-summary-flat.pdf

Life sciences study design

All studies must disclose on these points even when the disclosure is negative.

Sample size	This study randomized 52 patients with severe hypertriglyceridemia into three cohorts based on genotype: Cohort 1, familial chylomicronemia syndrome (FCS) patients with bi-allelic loss-of-function (LOF) lipoprotein lipase (LPL) pathway mutations; Cohort 2, multifactorial familial chylomicronemia syndrome (MCS) patients with heterozygous LOF LPL pathway mutations; Cohort 3, MCS patients without identified LPL pathway mutations. The primary endpoint was the mean intra-patient percent change in triglycerides from baseline following 12 weeks of evinacumab treatment in Cohort 3 patients. Exploratory objectives included an assessment of the effect of evinacumab versus placebo on the development of pancreatitis. Assuming a 20% drop out rate, a sample size 30 patients was considered adequate to analyze the percent change in triglycerides in the 3 genetic cohorts. To explore the effect of evinacumab on the development of pancreatitis, additional patients (up to a maximum of approximately 50 patients) were enrolled.
Data exclusions	There were no data exclusions.
Replication	As noted above, the study enrolled 52 patients, 51 of whom received study treatment during the double-blind treatment period. Patient data was analyzed according to treatment arm and by cohort at each study visit as described in the Methods section. Participant n numbers are provided in the text and figures, as appropriate.
Randomization	Fifty-two patients were randomized 2:1 (evinacumab:placebo) to receive intravenous evinacumab 15 mg/kg IV every 4 weeks (Q4W) or matching placebo Q4W for the 12-week double-blind treatment period, followed by a 12-week single-blind treatment period where all patients received evinacumab.
Blinding	Principal investigators, study site personnel and study patients were blinded to treatment during the 12-week double-blind treatment period. Blinding was not applicable for the 12-week single-blind treatment period.

Reporting for specific materials, systems and methods

We require information from authors about some types of materials, experimental systems and methods used in many studies. Here, indicate whether each material, system or method listed is relevant to your study. If you are not sure if a list item applies to your research, read the appropriate section before selecting a response.

Materials & experimental systems

n/a	Involved in the study
☐	☒ Antibodies
☒	☐ Eukaryotic cell lines
☒	☐ Palaeontology and archaeology
☒	☐ Animals and other organisms
☐	☒ Clinical data
☒	☐ Dual use research of concern

Methods

n/a	Involved in the study
☒	☐ ChIP-seq
☒	☐ Flow cytometry
☒	☐ MRI-based neuroimaging

Antibodies

Antibodies used	Evinacumab-dgnb
Validation	Evinacumab-dgnb is an angiopoietin-like protein 3 (ANGPTL3) inhibitor monoclonal antibody (IgG4 isotype) produced by recombinant DNA technology in Chinese hamster ovary (CHO) cell suspension culture. Evinacumab-dgnb has an approximate molecular weight of 146 kDa. For further information, please see the prescribing information, which may be found here: https://www.regeneron.com/downloads/evkeeza_pi.pdf

Clinical data

Policy information about clinical studies

All manuscripts should comply with the ICMJE guidelines for publication of clinical research and a completed CONSORT checklist must be included with all submissions.

Clinical trial registration	ClinicalTrials.gov NCT03452228, EudraCT 2016-003307-62
Study protocol	Qualified researchers may request access to the study protocol at https://vivli.org/.
Data collection	Patient data was collated in a clinical setting, and all study locations are detailed on ClinicalTrials.gov. The time period for recruitment and data collection ran from June 07, 2018 to July 23, 2020.<br><br>The study was conducted at 17 sites across four countries (Canada, 2 sites [Quebec]; Italy, 2 sites [Naples, Rome]; UK, 4 sites [Manchester, London, Birmingham]; USA, 9 sites [Philadelphia, Dallas, Pennsylvania, Kansas City, Milwaukee, Houston, New York, Atlanta, Boca Raton). Further details are provided in the Supplementary Information.
Outcomes	The pre-defined primary endpoint was percent triglyceride lowering from baseline following 12 weeks of evinacumab treatment in patients without LOF mutations in genes in the LPL pathway (Cohort 3).<br><br>The pre-defined secondary endpoints were:<br>1. Percent triglyceride lowering from baseline following 2 to 24 weeks of evinacumab treatment in the overall study population, and in subgroups with homozygous or compound heterozygous LOF LPL pathway mutations (Cohort 1), heterozygous LOF LPL pathway mutations (Cohort 2), and without identified LPL pathway mutations (Cohort 3).<br><br>2. Changes in patient reported abdominal and gastrointestinal symptoms, dietary habits, and symptom/dietary impact measures, assessed via questionnaires.<br><br>3. Degree of pancreatic injury/inflammation through fluorine-18-fluorodeoxyglucose positron emission tomography/computed tomography (18F-FDG-PET) imaging at baseline (placebo run-in period) and change from baseline following 12 weeks of treatment with evinacumab as assessed by 18F-FDG standardized uptake values.<br><br>4. Degree of pancreatic injury/inflammation through diffusion-weighted magnetic resonance imaging at baseline (placebo run-in period) and change from baseline following 12 and 24 weeks of treatment with evinacumab as assessed by the apparent diffusion coefficient.<br><br>5. The total evinacumab concentrations, total angiopoietin-like 3 (ANGPTL3) concentrations, and anti-evinacumab antibody concentrations during evinacumab treatment and follow-up periods. Serum samples were collated at specified time points for the determination of these parameters.<br><br>6. Incidence and severity of treatment-emergent adverse events, serious adverse events, laboratory abnormalities, and other safety variables in patients treated with evinacumab. Laboratory safety assessments were based on blood and urine samples collated at

specified time points. Adverse events were coded using version 22.0 of the Medical Dictionary for Regulatory Activities.
